# Does Mass Azithromycin Distribution Impact Child Growth and Nutrition in Niger? A Cluster-Randomized Trial

**DOI:** 10.1371/journal.pntd.0003128

**Published:** 2014-09-11

**Authors:** Abdou Amza, Sun N. Yu, Boubacar Kadri, Baido Nassirou, Nicole E. Stoller, Zhaoxia Zhou, Sheila K. West, Robin L. Bailey, Bruce D. Gaynor, Jeremy D. Keenan, Travis C. Porco, Thomas M. Lietman

**Affiliations:** 1 Programme FSS/Université Abdou Moumouni de Niamey, Programme National de Santé Oculaire, Niamey, Niger; 2 F.I. Proctor Foundation, University of California San Francisco, San Francisco, California, United States of America; 3 Dana Center for Preventive Ophthalmology, Wilmer Eye Institute, Johns Hopkins University, Baltimore, Maryland, United States of America; 4 Clinical Research Unit, Department of Infectious and Tropical Diseases, London School of Hygiene and Tropical Medicine, London, United Kingdom; 5 Department of Ophthalmology, University of California San Francisco, San Francisco, California, United States of America; 6 Department of Epidemiology & Biostatistics, University of California San Francisco, San Francisco, California, United States of America; 7 Institute for Global Health, University of California, San Francisco, California, United States of America; The Carter Center, United States of America

## Abstract

**Background:**

Antibiotic use on animals demonstrates improved growth regardless of whether or not there is clinical evidence of infectious disease. Antibiotics used for trachoma control may play an unintended benefit of improving child growth.

**Methodology:**

In this sub-study of a larger randomized controlled trial, we assess anthropometry of pre-school children in a community-randomized trial of mass oral azithromycin distributions for trachoma in Niger. We measured height, weight, and mid-upper arm circumference (MUAC) in 12 communities randomized to receive annual mass azithromycin treatment of everyone *versus* 12 communities randomized to receive biannual mass azithromycin treatments for children, 3 years after the initial mass treatment. We collected measurements in 1,034 children aged 6–60 months of age.

**Principal Findings:**

We found no difference in the prevalence of wasting among children in the 12 annually treated communities that received three mass azithromycin distributions compared to the 12 biannually treated communities that received six mass azithromycin distributions (odds ratio = 0.88, 95% confidence interval = 0.53 to 1.49).

**Conclusions/Significance:**

We were unable to demonstrate a statistically significant difference in stunting, underweight, and low MUAC of pre-school children in communities randomized to annual mass azithromycin treatment or biannual mass azithromycin treatment. The role of antibiotics on child growth and nutrition remains unclear, but larger studies and longitudinal trials may help determine any association.

## Author Summary

Recent studies suggest that antibiotic use could have an effect on growth in humans. Azithromycin is an antibiotic used for trachoma control, and hence, may have an unintended benefit of improving child growth. Niger is a trachoma-endemic country where mass antibiotic distributions for trachoma take place and where malnutrition is widespread among children. In addition, azithromycin may have an effect on common childhood diseases associated with malnutrition, such as diarrhea, pneumonia, and malaria. In a community-randomized trachoma trial in Matameye, Niger, we assessed child growth by measuring height, weight, and mid-upper arm circumference of pre-school children who have received 3 years of annual or biannual mass azithromycin treatment. While these measures were better in the biannually treated communities, the difference was not statistically significant. Thus, further research will help determine the impact of antibiotics on child growth and nutrition.

## Introduction

Non-specific antibiotic use has been employed to enhance weight gain in livestock since the 1950s [1]. Previous studies have examined the association between antibiotics and livestock [1]–[6]. These studies have identified benefits of using antimicrobials to improve animal growth, prevent and treat infections, and enhance feed efficiency [7]. In Africa, studies have investigated the effect of antibiotics to prevent and treat disease outbreaks among animals [8]. Anti-parasitic agents have been shown to increase weight in humans, presumably due to their effect on soil-transmitted helminthes [5]. Childhood illnesses such as diarrhea, pneumonia, and malaria have been linked to malnutrition [9].

The World Health Organization (WHO) recommends repeated community-wide oral azithromycin distribution for the control of blinding trachoma. Azithromycin is effective against the ocular strains of chlamydia that cause trachoma but may also have an effect on common childhood diseases associated with malnutrition, such as diarrhea, pneumonia, and malaria. Undernutrition is typical in the trachoma-endemic areas where these antibiotic distributions take place. For example, approximately half of Nigerien children under-5 years of age live with chronic malnutrition and one in 10 face severe acute malnutrition [10], [11]. Here, we collect anthropometric measurements from pre-school children in a community-randomized trial of mass oral azithromycin distributions for trachoma in Matameye, Niger. We compare height, weight, and MUAC in communities that have received 3 years of mass azithromycin treatments: annual *versu*s biannual (twice-yearly). We hypothesize that anthropometric indices are improved in children who are randomized to receive more treatments.

## Methods

### Ethics Statement

The study obtained ethical approval from the University of California, San Francisco Committee for Human Research and the Comité d'Ethique du Niger (the Ethical Committee of Niger). This study is registered at ClinicalTrials.gov, number NCT00792922. The study was carried out in accordance with the Declaration of Helsinki. Verbal consent was obtained from the local chiefs of each community before randomization. Verbal informed consent from each child participant's guardian was obtained prior to the examination. This consent process was appropriate given the high rates of illiteracy in the study area and was approved by all institutional review boards.

### Study Setting and Design

The Partnership for the Rapid Elimination of Trachoma (PRET) is a cluster-randomized clinical trial (clinicaltrials.gov trial, NCT00792922) [12] in the Matameye district of the Zinder region in Niger. A 2×2 factorial design was used to assess the effects of standard (80%) and enhanced coverage (90%) of annual mass azithromycin treatment of everyone *versus* biannual mass azithromycin treatment of children (6 months to 12 years) for trachoma and infection. Each cluster, referred to as a *community* in this manuscript, is in a government health unit from one of six health centers (Centres de Santé Intégrée, CSI).

### Community Randomization

Within each CSI, we conducted stratified blocked randomization of communities by high or low prevalence of clinical trachoma prevalence in children to account for community-level predictors in study arms prior to treatment. As previously described, communities were randomized to treatment arms, and individual participants were randomly selected from communities for trachoma monitoring [13]. Inclusion criteria for the PRET communities in Matameye were: population between 250–600 at the previous census, and ≥10% prevalence of active trachoma among children (<60 months of age) (trachomatous inflammation - follicular [TF] and/or trachomatous inflammation – intense [TI] per WHO trachoma grading system) [14]. Of 235 communities in the Zinder region of Niger, 72 communities were eligible for the PRET study. 48 were randomly selected for the PRET study. TCP generated the random allocation sequence of clusters using the statistical package R (version 2.12; R Foundation for Statistical Computing, Vienna, Austria; www.r-project.org) [15] by TCP [13]. Individuals were randomly selected for trachoma monitoring using MS Access (v2007) by study staff. Study staff enrolled and assigned clusters to interventions.

### Community and Individual Randomization for Substudy

In this substudy, we collected anthropometric measurements from pre-school children from 12 communities randomized to annual treatment of everyone and 12 communities randomized to biannual treatment of children during the 36-month study visit in August/September 2013. From the PRET study census in May 2013, individuals were randomly selected for anthropometry using the statistical package R prior to field data collection [15] by TCP [13].

Annually treated communities received three rounds of mass azithromycin treatment. Biannually treated communities received six rounds of mass azithromycin treatment. In annually treated communities, participants aged 6 months and older received a directly observed dose of oral azithromycin (1 mg/kg with a maximum dose of 1 gm). Study participants in biannually treated communities aged 6 months to 12 years were offered oral azithromycin. In both study arms, those less than 6 months of age were offered topical tetracycline ointment (1%) applied to both eyes twice a day for 6 weeks.

Our goal was to collect anthropometric measurements of 50 children in each community. To that end, 62 children, aged 6–60 months at the time of this sub-study, were randomly selected from the randomized registration lists generated from the follow-up PRET study census. Thus, these children were under 30 months of age at the PRET baseline census. Anthropometric measurements were collected at a centralized exam station in each community. If there were less than 50 children in the community, then anthropometric measurements were collected for all children.

Prior to the 36-month study visit, four individuals from the Niger Ministry of Health participated in a 1-day interactive WHO anthropometry training [16] in Niamey, Niger led by the F.I. Proctor Foundation team (University of California at San Francisco). In a previous study in Ethiopia, we demonstrated reproducibility of anthropometric measurements among team members who participated in our training [17]. Two of the four trainees had previous experience in anthropometry. The training curriculum included measuring recumbent length, standing height, weight, and MUAC.

For children younger than 2 years of age, we measured recumbent length; standing height was measured for children older than 2 years (Schorrboard; Schorr Productions, LLC, Olney, MD). Height and length were measured to the nearest 0.1 cm. For children who were unable to stand on their own due to sickness or weakness, we measured recumbent length and subtracted 0.7 cm for an estimated height per the WHO conversion formula [18]. Children were weighed individually with little or no clothing; if necessary, children were weighed while being held by a parent or guardian using the taring function (seca 874 flat floor scale; seca GMBH & Co. Kg, Hamburg, Germany). Trained anthropometrists ensured the scale was on a flat surface. Weight was measured to the nearest 0.1 kg. MUAC was measured to the nearest 0.1 cm with non-stretchable measuring tape developed by Johns Hopkins University [19]. Measurements were collected in triplicate. Children with severe malnutrition or illness were referred to local health posts for treatment. Anthropometrists recorded data on paper forms and sent to San Francisco for data entry. They were masked to treatment allocation and antibiotic coverage data. Community members were not masked to treatment allocation.

### Outcome Variables

For our outcome measurements to assess the difference in anthropometric measurements of pre-school children in communities randomized to annual or biannual mass azithromycin treatment, we used the WHO Anthro R macros [20] based on the 2006 WHO child growth standards [21] and converted anthropometric measurements: age- and sex-adjusted community-level nutritional Z scores for wasting (weight-for-height Z score [WHZ]), low MUAC (MUAC Z score [MUACZ]), stunting (height-for-age Z score [HAZ]), and underweight (weight-for-age Z score [WAZ]). We defined low anthropometric scores as Z<−2.0 (based on the WHO reference population) [22]. For each of the following variables, we used the indicated cutoffs to produce a binary outcome variable: wasting, stunting, underweight, severe underweight (Z<−3), and low MUAC.

### Analytic Approach

For the binary variables, we used clustered logistic regression with CSI and community as random effects, and treatment arm as a fixed effect predictor. We used the log-link to yield estimates for the odds ratios. We also analyzed anthropometric measurements as continuous outcomes using the Wilcoxon signed rank test to compare community means. In addition, we report the pseudomedian (Hodges-Lehmann estimator) for these means by arm. In all communities, inclusion was restricted to participants who took their assigned treatment. Overall, 1,034 individuals were included in this analysis.

As a secondary analysis, for WHZ scores, we only included child participants in this sub-study who received their assigned study treatment: annual (three times) or biannual (six times). To confirm this, we included participants who received their assigned treatment in the annual communities for WHZ scores.

### Sample Size

We estimated that 24 communities (12 communities per group) would provide greater than 80% power to detect an absolute difference of 6% wasting between the two arms, assuming 50 children per community, 2-sided α = 0.05 and an intraclass correlation coefficient (ICC) of 0.015 [23] and a prevalence of wasting of 8%. For all statistical analyses, we used the statistical CI package STATA 10 (Stata Corp., College Station, TX) [15]. For this single cross-sectional visit comparison, there were no interim analyses or stopping guidelines.

## Results

**Figure 1:**
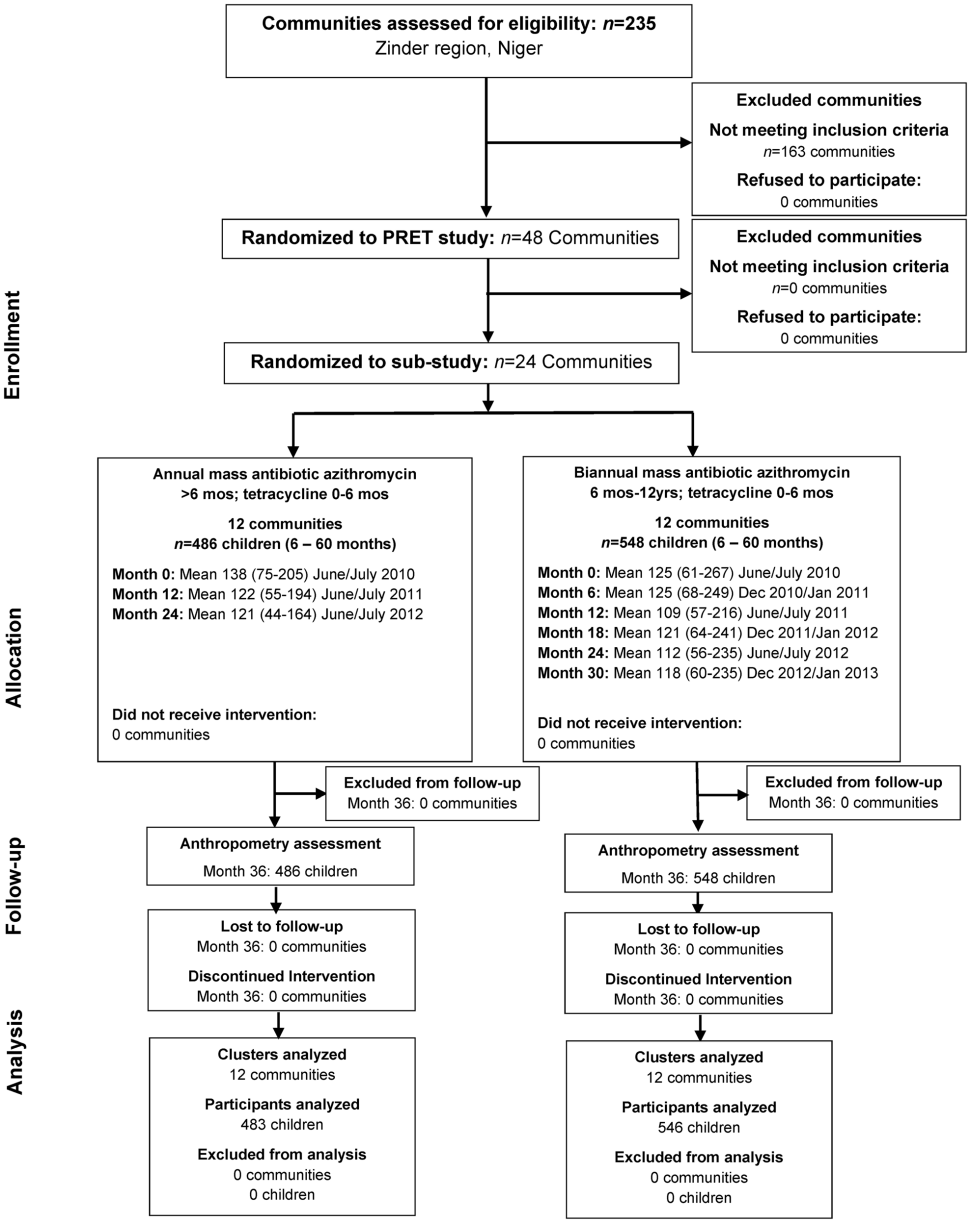
Participant flow. For the PRET study, 235 communities were assessed in the Zinder region of Niger. 72 communities met the inclusion criteria. Of eligible communities, 48 were randomized to the PRET study. A total of 24 communities were enrolled in this substudy, with 12 communities randomized to annual treatment and 12 communities randomized to biannual treatment according to the study design. All 24 communities remained in this substudy. This figure summarizes the mean number of children aged 6–60 months at each time point by study arm. The children for the anthropometry assessment are the number of children (target age) present at month-36.

All 24 communities remained in this substudy (Figure 1). In the PRET study, the 12 annually treated communities received treatment three times (June/July 2010, June/July 2011, and June/July 2012). The 12 biannually treated communities were treated six times (June/July 2010, December 2010/January 2011, June/July 2011, December 2011/January 2012, June/July 2012, and December 2012/January 2013). We collected anthropometric measurements from a total of 1,034 children in 24 communities (486 annual treatment arm; 548 biannual treatment arm). The confidence intervals (CI) for antibiotic coverages among children in the 12 annual communities are 93.8–96.5, 88.4–94.6, and 87.5–94.1. In the 12 biannual communities, the CI for antibiotic coverages among children were 92.4–95.9, 90.8–96.5, 91.9–95.6, 87.1–96.3, 87.3–91.3, and 87.5–94.1. We collected anthropometric measurements in August and September 2013, which was more than 1 year after the last treatment for the annually treated communities and about 8 months after the last treatment for the biannually treated communities. As shown in Table 1, the baseline characteristics of children who were eligible for inclusion in this study (includes ≤30 months of age for follow-up 3 years post-baseline) are comparable between treatment arms. All study communities were treated per study protocol and no communities were lost to follow-up. There were no serious adverse events reported for the study medication.

**Table 1:** Baseline characteristics of 24 communities randomized (1∶1) to annual or biannual mass azithromycin treatments in a cluster randomized clinical trial for trachoma in Niger.

	Mean (95% CI or range where specified)		
	Annual treatment (12 communities)	Biannual treatment (12 communities)	p-value**
Children per community ≤30 months	72 (range 37–119)	66 (range 36–124)	0.66
Age of children (months)	18.4 (17.3–19.4)	18.7 (17.4–19.9)	0.91
Proportion female, %	52.1% (49.3–54.8)	49.0% (45.1–52.9)	0.22
Prevalence trachoma TF*	23.4% (14.9–32.0)	17.6% (12.5–22.7)	0.22
Prevalence of trachoma TI*	7.1% (1.0–13.3)	5.0% (1.9–8.2)	0.91

*TF, trachomatous inflammation - follicular; TI, trachomatous inflammation – intense, both from a random sample of children aged ≤30 months of age.

**p-values: All Wilcoxon rank-sum except linear mixed effects regression for age of children.

In the biannually treated communities and the annually treated communities (mixed effects logistic regression with community and CSI as a random effect), the odds ratio for wasting in the biannually treated communities relative to the annually treated communities was 0.89 (95% CI = 0.53 to 1.49), odds ratio for stunting was 0.78 (95% CI = 0.54 to 1.13), and odds ratio for underweight was 0.88 (95% CI = 0.66 to 1.19). When restricted to ages 6 to 24 months, the odds ratio for stunting was 0.85 (95% CI = 0.51 to 1.42). Among children under 5 years of age, the odds ratio for MUAC was 0.62 (95% = 0.32 to 1.17). In the annually and biannually treated communities, the mean prevalence of severe wasting (WHZ<−3.0) was not significantly different (OR = 0.85, 95% CI = 0.34 to 2.12). The estimated ICC was 0.024 (95% CI = 0.019 to 0.077, bootstrap percentile) for height and 0.008 (95% CI = 0.007 to 0.060, bootstrap percentile) for weight. As shown in Tables 2 and 3, we were unable to demonstrate a significant difference in anthropometric measurements among children from annually treated communities and biannually treated communities.

**Table 2:** Wasting, low MUAC, stunting, and underweight in children aged 6–60 months from 24 communities randomized (1∶1) to annual or biannual mass azithromycin treatment.

	Annual treatment (12 communities)		Biannual treatment (12 communities)			
Measurement	%	No./total	%	No./total	Odds ratio (95%CI)*	p-value
Wasting	13.9	66/475	12.8	62/538	0.89 (0.53 to 1.49)	0.64
Low MUAC	17.4	66/379	12.0	52/435	0.62 (0.32 to 1.17)	0.14
Stunting	59.0	285/483	52.9	289/546	0.78 (0.54 to 1.13)	0.20
Underweight	44.1	213/483	41.2	225/546	0.88 (0.66 to 1.19)	0.41

*Mixed effects logistic regression with community as a random effect. All measurements are based on Z score<−2.0. Numbers may be different because of some loss during field examination.

MUAC: mid-upper arm circumference.

**Table 3:** Anthropometric Z-scores in children aged 6–60 months from 24 communities randomized (1∶1) to annual or biannual mass azithromycin treatment.

	Annual		Biannual			
Metric	Pseudo- median	95% CI	Pseudo- median	95% CI	Estimated Difference (95%CI)*	p-value
WHZ	−0.76	−0.98 to −0.59	−0.76	−0.96 to −0.35	0.08 (−0.24 to 0.46)	0.68
MUACZ	−1.03	−1.22 to −0.67	−0.91	−1.06 to −0.75	0.09 (−0.31 to 0.41)	0.68
HAZ	−2.32	−2.56 to −1.81	−1.98	−2.42 to −1.29	0.27 (−0.19 to 0.88)	0.27
WAZ	−1.88	−2.04 to −1.63	−1.62	−1.86 to −1.33	0.23 (−0.05 to 0.56)	0.09

*Pseudomedian (Hodges-Lehmann estimator) difference between the biannual arm and annual arm. Positive values correspond to larger measurements in the biannual arm.

WHZ: weight-for-height z-score.

MUACZ: mid-upper arm circumference z-score.

HAZ: height-for-age z-score.

WAZ: weight-for-age z-score.

## Discussion

Our community-randomized clinical trial demonstrated no significant difference in anthropometric measurements of pre-school children from communities randomized to annual mass azithromycin treatments of everyone in the community *versus* biannual mass azithromycin treatments of children only. Therefore, not only was the frequency of mass treatments different between the study arms, but so were the populations treated. While pre-school children in annually treated communities had a higher prevalence of wasting, stunting, low MUAC, and underweight in comparison to biannually treated communities, these differences were not statistically significant.

Our study has some important limitations worthy of discussion. First, we examined the effects of mass azithromycin distributions on children who received annual treatment and biannual treatment. However, if we examined children from communities who received no antibiotics and children from communities who received many treatments, differences in child growth and nutrition might have been more apparent. In addition, any effect on wasting, if present at all, might be more likely to be observed closer to the time of the antibiotic distribution. Second, this study was a subset of communities from a larger trial; perhaps a larger study could detect a significant difference. Future studies could be powered to detect a smaller effect on height and weight. Third, the study design was post-test only (no baseline data were collected) and we did not collect longitudinal data. The inclusion of baseline data can sometimes improve power, if using a change score or using baseline as a covariate predicting final outcome. Note however that the majority of 0–5 year-olds at the end of the study had not been born at the start of the study. The randomized post-test design does permit valid inference, since the treatment assignments are stochastically independent of any other explanatory covariate (including baseline anthropometry) [24]. In addition, this was in an area meso-endemic for trachoma, which may be a sign of poor socioeconomic conditions. It is unclear what, if any, effect would have been seen in poorer or wealthier areas.

Finally, it may be difficult to assess the true effect of antibiotics on anthropometry due to a possible seasonal effect in this local context. These measurements were collected towards the end of August and September, near the end of the rainy season and before the harvest, coinciding with Niger's season of rains, hunger, and malaria (June to October). During this time, the rate of acute malnutrition in children under 5 years of age is high, surpassing the global emergency threshold for malnutrition [10]. Antibiotics might not be expected to affect acute malnutrition–indeed we find no significant difference in wasting between the two arms. Children also become more susceptible to other infectious diseases such as malaria, acute respiratory diseases, and diarrheal diseases among other infections [10]. The burden of malnutrition and malaria is detrimental to child health. In Niger, malaria is the leading cause of death among children under 5 years of age and pregnant women [10]. These factors may make it more challenging to detect an effect of azithromycin on child growth and nutrition.

A recent cluster-randomized trial in the same study area of Niger, but in different communities, did not find significant difference in anthropometric measurements due to mass antibiotic treatments at 1 year [25]. Children from communities randomized to two azithromycin treatments had higher anthropometry indices compared to children from communities randomized to only one, although differences were not statistically significant.

There are positive and negative effects of the mass oral azithromycin distributions. Mass antibiotic distributions have been successful for trachoma control and azithromycin is well tolerated [26]. Studies demonstrate that azithromycin may be beneficial for infectious diseases such as pneumonia [27], diarrhea [28], [29], and malaria [30]. In addition, previous case-control [31] and cluster-randomized [32] trials in Ethiopia found significant reductions in childhood mortality with mass azithromycin distributions. Mass antibiotic distribution programs also have the potential to select for antibiotic resistance. Distributions have been proven to select for nasophargyngeal pneumococcus [33], although resistance decreased when mass treatments were discontinued [34]. In addition, there is potential for azithromycin to affect the proposed pathway from small intestinal mucosal damage to growth faltering in two ways: (*i*) it may reduce or modulate the intestinal microbial load, thereby reducing microbial translocation and/or (*ii*) as a macrolide, it may directly reduce systemic immune activation via its well-recognized immunomodulatory effects [35].

In conclusion, we did not find a significant difference in height, weight, and MUAC of pre-school children in communities randomized to annual mass azithromycin treatment *versus* biannual mass azithromycin treatment, and cannot support a role of antibiotics on child growth and nutrition. Additional studies are needed to further explore the potential impact of antibiotics on child growth. If antibiotics do enhance child growth and nutrition, this could significantly reduce infant and child mortality worldwide.

## Supporting Information

Text S1 Trial protocol.
(PDF)

Text S2 Consort checklist.
(PDF)
