# Supplementary material for: Does Mass Azithromycin Distribution Impact Child Growth and Nutrition in Niger? A Cluster-Randomized Trial
Source: PLoS Negl Trop Dis. 2014 Sep 11;8(9):e3128. doi: 10.1371/journal.pntd.0003128 (PMC4161345; doi:10.1371/journal.pntd.0003128)
Supplement: Text S1 — Trial protocol. (PDF) [file pntd.0003128.s001.pdf]

Partnership for the Rapid Elimination of Trachoma (PRET)

Manual of Operations and Procedures

© Johns Hopkins Medical Institutions
600 N Wolfe St Baltimore MD, 21205
Tel: (410) 955-2606
FAX: (410) 955-0096

Table of Contents

CHAPTER 1: OVERVIEW **6**

1.1 Overview **6**

1.2 Research Questions **6**

1.3 Overall Design **7**

CHAPTER 2: BACKGROUND AND SIGNIFICANCE **8**

2.1 Trachoma as a Public Health Problem **8**

2.2 Anticipated Impact **9**

CHAPTER 3: RANDOMIZATION ARMS AND SAMPLE SIZE **10**

3.1 Overview: **10**

3.2 Randomization Arms **10**

3.3 Communities and Sentinel Children **11**

3.4 Sample Size **11**
3.4.1 Villages 11

CHAPTER 4: ELIGIBILITY, CONSENT AND ENROLLMENT **13**

4.1 Eligible Communities **13**

4.2 Eligible Children **13**

4.3 Consent Procedures **13**

4.4 Risks and Efforts to Minimize Risk **14**

CHAPTER 5: PROCEDURES: CENSUS OF COMMUNITIES 15

5.1 Overview 15

5.2 Baseline Census.. 15

5.3 Interim Census 16

CHAPTER 6: PROCEDURES: STUDY VISITS.. 17
6.1 Overview 17

6.2 Baseline Visit.. 17
6.2.1 Trachoma Assessment 18
6.2.2 Laboratory Procedures 19
6.2.3 Shipping and Storage of Samples.. 21
6.2.4 Laboratory Processing Procedures.. 22
6.2.5 Photograph Grading.. 22
6.2.6 Follow-up of Children 23

6.3 Forms and Swab Management for Visits 23

6.4 Missed Visits... 24

6.5 Follow-Up Visits 24

6.6 Study Visit Sample Schedule 24

CHAPTER 7: INTERVENTION: MASS TREATMENT IMPLEMENTATION 25

7.1 Overview: Treatment with Azithromycin 25

7.2 Six monthly Mass Treatment.. 25

7.3 Monitoring Mass Treatment Coverage 26
7.3.1 Mass Treatment Verification Procedure.. 26

7.4 Masking Intervention 27

CHAPTER 8: COST EFFECTIVENESS DATA COLLECTION 28

8.1 Overview 28

8.2 Cost Data Collection 28

CHAPTER 9: STUDY INSTRUMENTS **29**

9.1 Overview **29**

9.2 Census Forms **29**
9.2.1 Baseline Census Form 29
9.2.2 Census Update Forms 32
9.2.3 Master List of Eligible Children for Survey 33

9.3 Ocular Form **33**

9.4 Photolog Book **34**

9.5 Ocular Specimen Shipping List **35**

9.6 Mass Treatment Log Book **35**

9.7 Cost **35**

CHAPTER 10: TRAINING AND CERTIFICATION **39**

10.1 Trachoma Grading **39**

10.2 Laboratory Specimen Handling **39**

10.3 Photography **40**

10.4 Study Forms **40**

10.5 Data entry **40**

CHAPTER 11: DATA MANAGEMENT AND ROUTINE REPORTS **42**

11.1 Overview **42**

11.2.1 Study Forms Preparation **42**
11.2.2 Daily Data Management Activities in Field 43
11.2.3 Data Entry 43
11.2.4 Preparation of Data For Transfer To UCSF 43
11.2.5 Preparation of Data For Transfer To LSHTM 44
11.2.6 Data Editing At the Coordinating Center 44
11.2.7 Data Management Process 44
11.2.8 Periodic Reports 45
11.2.9 PCR Result Reports 45
11.2.10 Data Storage 45

CHAPTER 12: QUALITY ASSURANCE POLICIES AND PROCEDURES 46
12.1 Overview 46
12.2 General Guidelines for Form Completion 47
12.3 Missing Data on Study Forms 47
12.4 Changing Responses on Study Forms 47
12.5 Oversight 48
12.6 Adherence to Procedures 48
12.7 Quality Assurance for Field Trachoma Grading 48
12.8 Quality Assurance for Specimens 49
CHAPTER 13: ADVERSE EVENTS 50
CHAPTER 14: ANCILLARY STUDIES 51
14.1 Risk of Re-emergence: Pediatric Longitudinal Study 51
14.2 Cost Effectiveness Study 51
14.3 Anthropometry: Niger 51
14.4 Blood Collection: Niger 52

Chapter

1

Chapter 1: Overview

1.1 Overview

The goal of this community-based randomized clinical trial is to test hypotheses about the impact on trachoma and ocular *C. trachomatis* infection of varying frequency and coverage of mass azithromycin use in communities. This trial will add to the evidence base to improve the implementation of the antibiotic component of the SAFE (Surgery/Antibiotics/ Face washing/Environmental change) strategy for trachoma control. Through the PRET partnership, we will address significant trachoma program barriers: slow decline in trachoma in these communities and re-emergence of infection following mass treatment.

The overall objective is to determine optimal strategies for elimination of trachoma and infection under differing mass treatment coverages. Specific objectives to meet this overall objective include:

- To determine the effective coverage (80% to 90% versus above 90% in children) of mass azithromycin treatment necessary for hyperendemic areas that will lead to rapid reduction of infection to less than 5%.

- To determine if mass azithromycin administration every six months in children can reduce infection in the entire community, and would be an effective control strategy in practice. In other words, do children act as a core group in the transmission of trachoma?

To meet the overall objective, then, we have two major activities: (1) Conduct clinical trials of communities in Niger of alternative coverages and frequencies of administration of mass antibiotic treatment; (2) Determine the cost-effectiveness of these different strategies from program perspective.

1.2 Research Questions

1) COVERAGE: What are the differences at three years post-baseline in rates of trachoma and ocular C. trachomatis infection in villages randomized to mass antibiotic coverage of 80% to 89% (as measured in children ages less than 10 years) compared to villages randomized to enhanced coverage of 90% or greater?

2) FREQUENCY OF MASS TREATMENT: What are the differences at three years post-baseline in rates of trachoma and ocular C. trachomatis infection in hyper-endemic villages in Niger randomized to mass treatment of children ages twelve and under biannually (every six months) compared to yearly mass treatment of everyone?

1.3 Overall Design

We propose a community-randomized design within Niger which considers hyperendemicity to address questions of coverage and frequency. Within these study areas, communities will be randomized to two different coverage targets (80%-89% versus ≥90%) for three years of mass treatment. We will further randomize communities to annual mass azithromycin distribution versus every 6-month distribution. Only children ages 12 and under will receive azithromycin treatment. We will evaluate the efficacy of guiding further mass treatment according to a laboratory test for Chlamydia or WHO guidelines.

Sentinel children randomly selected as cross sectional samples in these communities will be monitored at baseline, 6, 12, 18, 24, 30, and 36 months for infection and clinical disease. The three-year study is in accord with the WHO guidelines for three years of mass treatment followed by a re-survey to determine areas in need of further treatment.

Primary analyses will focus on the comparison at three years of trachoma and infection rates in villages randomized to 90% or greater coverage versus less than 90%, and in villages randomized to yearly treatment of everyone versus 6 monthly treatment of children. Primary analyses will consider the 2 X 2 factorial design, the baseline prevalence, and baseline indicators of environmental hygiene as co-variates and effect modifiers. Secondary analyses will evaluate trachoma and infection in children, adjusting for clustering at the village level.

There are two ancillary studies nested within the trial. First, we will determine the cost-effectiveness of the different treatment coverage and administration frequency arms. The data on costs associated with a lab-based strategy to guide mass treatment cessation will assist in development of the cost of such a tool if it is finally developed. Moreover, we must weigh the potential additional costs associated with treatments every six months for children, or higher coverage in villages, against the benefit in terms of reduction of trachoma infection.

The second study will determine rates of re-emergence and the effect of multiple rounds of mass treatment within children. In communities selected at random within each arm, we will follow all children ages 9 years and under longitudinally after mass treatment for re-emergent trachoma infection and clinical disease. We will determine risk factors predicting re-emergence.

These studies provide significant value to the trial in helping elucidate treatment "failures", in providing cost-effectiveness data, as well as effectiveness data, on the treatment strategy alternatives.

Chapter

2

Chapter 2: Background and Significance

2.1 Trachoma as a Public Health Problem

Trachoma, caused by ocular *Chlamydia trachomatis,* is the leading infectious cause of blindness worldwide. There are profound inequalities in the burden of trachoma, as it affects almost exclusively the poorest countries in the world, as measured by World Bank PovCal indices. Within affected countries, the geographic burden of trachoma is highest in relatively rural, poor regions. Research has shown that within these regions, trachoma disproportionately affects the most poor, and those who traditionally have no voice in leadership: the pool of trachoma in these impoverished communities is greatest in children less than five years of age, and women develop blindness from trachoma at a rate 2–3 times that of men.

In addition to the heavy personal and societal burden inflicted by loss of vision, there are significant economic impacts as well. The 3.8 million cases of blindness and 5.3 million cases of low vision due to trachoma are estimated to diminish productivity by $2.9 billion each year[1].

In recognition of the public health impact of trachoma, the WHO established the Global Alliance to Eliminate blinding Trachoma by 2020 (GET 2020). A multi-faceted strategy to control all phases of trachoma has been endorsed by the WHO. It consists of **S**urgery (to repair lids distorted by trachoma (trichiasis) in imminent danger of vision loss), **A**ntibiotics (mass antibiotic treatment to reduce the community pool of infection with *Chlamydia trachomatis*), **F**ace washing (to reduce transmission from ocular and nasal secretions), and **E**nvironmental improvements (to interrupt transmission and prevent re-emergence). With even partial implementation of the SAFE strategy to 80% of those who need it, it is estimated that over 15 million DALYs per year globally would be saved[2].

However, the trajectory for elimination of trachoma in these countries has been disappointingly slow. How long and with what frequency must we mass treat communities with antibiotics for active trachoma to achieve elimination? While WHO has recommended mass antibiotic treatment coverage targets of 80%, there are no solid data on how high the coverage should be and the frequency of administration. High antibiotic treatment coverage rates, and

---

[1] Frick KD, Basilion EV, Hanson CL, Colchero MA Estimating the burden and economic impact of trachomatous visual loss. Ophthalmic Epidemiol. 2003 (2):121-32

[2] Baltussen RM, Sylla M, Frick KD, Mariotti SP Cost-effectiveness of trachoma control in seven world regions. Ophthalmic Epidemiol. 2005;:91-101

repeated treatments, may eliminate infection in some communities, but with almost a three to five year lag time between reduction in infection and significant reduction in the clinical signs of trachoma, communities remain targets of resources for mass treatment even when such resources may be better-deployed elsewhere[3]. Even with high coverage, some treated children with high loads of infection continue to have infection post treatment, arguing for approaches to targeted surveillance or more frequent coverage[4]. With renewed interest in trachoma as a "neglected tropical disease", and the possibility where ecologically justified of integrated programs, there is an even more urgent need to clarify how best to use azithromycin.

The consortium brings together the leading trachoma research teams, at Johns Hopkins University (JHU), the London School of Hygiene and Tropical Medicine (LSHTM), and the University of California at San Francisco (UCSF). Consortium partners include Pfizer, who have pledged antibiotics for this project, advocacy partners including WHO and the Trachoma Control Programs in Tanzania, Niger, and The Gambia.

2.2 Anticipated Impact

Working with Ministries of Health and Niger's country programs, this trial of mass antibiotic intervention has the potential to alter the trajectory of blinding trachoma dramatically. This includes areas of hyperendemicity where programs have not yet started on a large scale as they have in other parts of the country. No other research partnership has the breadth and ability to undertake such a comprehensive, and critical, proposal for trachoma control. We have proposed the dissemination of results in a format for country National Trachoma Control programs and Ministry of Health, the ultimate end users who need this evidence base to drive improvements in implementation of SAFE for trachoma elimination.

[3] Solomon AW, Holland MJ, Alexander ND, Massae PA, Aguirre A, Natividad-Sancho A, Molina S, Safari S, Shao JF, Courtright P, Peeling RW, West SK, Bailey RL, Foster A, Mabey DC.Mass treatment with single-dose azithromycin for trachoma.N Engl J Med. 2004;351:1962-71

[4] West SK, Munoz B, Mkocha H, Holland MJ, Solomon AW, Foster A, Bailey RL, Mabey DCW. Infection with *Chlamydia trachomatis* after mass treatment of a trachoma hyperendemic community in Tanzania: a longitudinal study. Lancet 2005;366:1296-1300

Chapter

3

Chapter 3: Randomization Arms and Sample Size

3.1 Overview:

We propose to randomize communities in a 2 X 2 factorial design within Niger. Communities will be randomized (12 in each arm) to two different coverage targets (80%-89% versus ≥90%) for three years of mass treatment. Communities will be further randomized to annual mass azithromycin distribution for everyone versus every 6 month distribution in children ages twelve and under.

Sentinel children age five years and younger will be randomly selected from each community based on census of the community, as cross sectional samples of the rates of trachoma and infection. The census will be updated each year, and villages monitored at baseline, 6, 12, 18, 24, 30, and 36 months for infection and clinical disease. The three-year study is in accord with the WHO guidelines for three years of annual mass treatment followed by a re-survey to determine need for further treatment.

3.2 Randomization Arms

The basic structure of the trial is shown in the table below. In this 2 X 2 factorial design, communities will be randomized into four treatment arms. All communities will receive mass treatment with azithromycin at baseline. At present, recommended coverage of the community is 80% or better. We propose to evaluate the effect of two coverage targets, (estimated in children age 0 to 9 years old). The two arms would have 1:1 randomization at community level to one of two coverage targets: 80% to 89% coverage (reasonable program targets) and ≥90% (enhanced program coverage, requiring additional time and resources). Niger will be participating in the randomization arms for coverage, in order to determine the incremental effectiveness of extra effort in coverage in reducing infection and disease.

	80-90% Antibiotic coverage target	>90% Antibiotic coverage target
Annual azithromycin to everyone	Arm A	Arm B
Biannual azithromycin to children	Arm C	Arm D

The second component of the antibiotic trial is frequency of administration of mass treatment. We propose two frequencies of mass antibiotic administration, yearly for everyone in the community and every six months for children ages twelve and under.

3.3 Communities and Sentinel Children

In order to determine the impact of our treatment arms on trachoma and infection, we will randomly sample a sentinel group of up to 100 children within randomly selected communities. *We define communities as the smallest population unit for which health services are organized and trachoma control programs are implemented.* In Niger we will use villages comprised of 250-600 people. As described below, we plan to have 12 communities in each cell of the Niger arms. We need multiple communities to have a stable estimate of the impact of the treatment arm.

In Niger, 48 grappes (villages) of population size 250-600 people and approximately 50-100 children in the eligible age range cover the district of interest.

For this trial, we propose to monitor the changes in prevalence and infection over time according to treatment arm. For this, simple cross sectional samples of 50-100 children ages 0 to 5 years within the community at specified times are sufficient. This sample will enable us to determine the effect of treatment after each round. This sample will not be followed longitudinally, and at each sampling time, a new random sample will be drawn. To support the sampling, a census update will be performed yearly.

3.4 Sample Size

3.4.1 Villages

We estimated the sample size requirements for our two-by-two factorial design using simulation. Briefly, we generated simulated data assuming the alternative hypothesis and determined the sample size needed for how often (selecting 80%, our desired power) our statistical test would yield a rejection of the null hypothesis. We adopt a non-inferiority design[5] for each factor with non-inferiority limits of 8%. Assuming a standard deviation of 0.05 within each arm, a correlation of 0.5 between baseline and 36 month results (derived from previous studies), and no interaction between factors, we estimate that a total of 32 communities provides greater than 80% power for each main effect (95% one-sided non-inferiority). Smaller community sizes imply a higher variance. Conservatively, we assumed the variance was one third higher. Thus, to obtain greater than 80% power, we require 12 villages in each arm (for a total of 48 villages, again assuming a one-sided 95% non-inferiority comparison with limit 0.08).

We show the sample size for each hypothesis and assumption, recognizing the simple approach requested is not the analytic plan, and show that the simulation approach yields comparable estimates, and which includes consideration for our approach to analyses.

1) What are differences at 3 years post baseline in rates of trachoma and ocular *Chlamydia trachomatis* in communities randomized to mass antibiotic coverage of low (80-89%) versus high (>90%) coverage.

---

[5] Chow SC, Liu JP. Design and Analysis of Clinical Trials, 2d ed. New York, John Wiley, 2004

We hypothesize that higher coverage (as measured in ages <10 years) will result in lower prevalence of infection at three years in Niger.

In principle, this could be addressed by a two-sample T-test. We estimate that the inclusion of 12 communities per arm in Niger will provide greater than 80% power to detect a 6% difference in the prevalence of infection in individuals 0 to 5 years of age at 36 months, assuming a standard deviation of 5.0% and a two-tailed alpha of 0.05.

We have used our judgment to propose that a 6% difference in the prevalence of infection is the clinically relevant difference we wish to detect. After three years of mass treatment, we expect infection rates to be low, and the difference in terms of re-emergence between, for example, 2% and 7% is insignificant. Similarly, re-emergence rates may be higher, but we do not expect differences in the rates, if at three years infection rates are 10% to 15%.

2) What are the differences at 3 years post baseline in the rate of ocular *C.trachomatis* in communities randomized to mass antibiotic treatment of children every 6 months versus yearly treatment of everyone?

We hypothesize that treatment of children every six months will result in a lower prevalence of trachoma, infection at three years in Niger compared to yearly treatment of all residents.

As with Hypothesis 1, this could be addressed by a two-sample T-test. We estimate that the inclusion of 12 communities per arm in Niger will provide greater than 80% power to detect a 6% difference in the prevalence of infection in individuals 0 to 5 years of age at 36 months, assuming a standard deviation of 5.0% and a two-tailed alpha of 0.05. This computation was conducted using the same computation as for Hypothesis 1.

Chapter

4

Chapter 4: Eligibility, Consent and Enrollment

4.1 Eligible Communities

This study aims to determine the impact of different approaches to mass treatment on prevalence of trachoma and infection in villages that are hyperendemic for trachoma. Eligible communities are described below:

To be eligible for the trial in Niger, a community must meet the following criteria:

1. The community must be located in the target district around Matameye
2. The village chief consents to participation in the trial (this does not obviate the need for individual consent, but without overall leadership consent, the community as a whole cannot be part of the trial)
3. Communities must be more than four kilometers away from the center of any semi-urban areas, which are thought to have a lower prevalence of trachoma
4. Communities must have an estimated population of between 250-600 people.

4.2 Eligible Children

Within each community, we will monitor the following children in all 48 grappes:

1. The child is age 5 years and younger
2. The child must be a resident in an eligible, sample community (defined as either living in the community since birth, or moved in with parents or guardians).
3. The child must not have an ocular condition that would preclude grading trachoma or taking an ocular specimen.
4. The child must have an identifiable guardian capable of providing consent to participate.

Note that in 2 grappes of each of the 4 arms, we will monitor all 0-9 year-old children.

4.3 Consent Procedures

Several levels of recruitment are involved when working in Niger. At the community level our research team discusses the project with local leaders. This information is then imparted throughout the community where the research project and participation is discussed. Study personnel will address specific questions the community may have before allowing us entry.

Once leadership consents to participation, we will undertake a complete census of study areas by going house to house in each community. At this second level, we ask permission of the head of the household or an adult dwelling in the household to collect data on household characteristics and the age and gender of each resident of the household. At this time, we explain the following: the census will be used to monitor the uptake of mass treatment in the community and to draw a random sample of children ages 0-5. These children may be eligible to be in the surveys that will be done at baseline, and every six months after mass treatment. At this point we describe what the survey entails (trachoma assessment of the child, and a swab taken of the upper eye lid to detect chlamydia, the bug that causes trachoma). We ask them to consider that, if the household contains a child of eligible age who is selected, we will return to seek permission to examine the child.

Local team members will provide information and seek consent. The study coordinator will train the team in research ethics and informed consent. The team will explain that the purpose of the project is to determine severity of trachoma and infection before and after treatment by examining a sample of the children in the village. They will demonstrate the use of the swab and explain that participation is completely voluntary and will not affect access to health care or access to the mass treatment to come after the survey. Additionally, they will explain that different villages will receive different treatments in an effort to determine the best treatment, and that treatment for trachoma will be provided to everyone in the village once examinations are completed.

4.4 Risks and Efforts to Minimize Risk

There are minimal risks to the participants. Flipping the eyelid is only slightly uncomfortable and most children are not bothered by the procedure (in fact, many children can do it themselves). The swab is uncomfortable, and can be a painful procedure if the eye is very inflamed. However, the procedure does not harm the child, other than upset them for a few moments if it is uncomfortable. By informing the parents about the clinical status of the child, and motivating them to seek treatment, the assessment is actually helpful. There is no alternative to the clinical examination for determining trachoma status.

Procedures for protection against risk include the following: we are careful to have a single individual flip the lid while the senior lab technician carries out the examination, takes the swab, and the photograph of the upper eye lid. In this way, any psychological trauma to the child, or discomfort from having the eyelid flipped is minimized as the lab technician can work quickly. In addition, by having the person hold the eyelid, the swab procedure can be done safely without worry of accidental corneal scratch. We have never had an accident in all the years we have taken swabs for trachoma using these procedures. In the unlikely event of a corneal scratch, the team has an eye patch and topical tetracycline and can treat the scratch immediately. The results of the examination are told to the parents immediately after the examination, and not revealed to other members of the village.

The forms are stored in a folder, which after data entry, is kept in a locked file room. The data are transmitted to the main data centers without names, just identification numbers. Such procedures ensure confidentiality.

Chapter

5

Chapter 5: Procedures: Census of communities

5.1 Overview

The census is a critical piece of data collection, as it is the document by which we measure coverage of mass treatment in the community, and it is the sampling frame for drawing a random sample of children in each community. Once the communities have been selected, and agree to participate, the survey teams will then conduct a full census.

In the context of this trial, as described earlier, the community is a village (population of 250-500 people).

5.2 Baseline Census

The census team will meet with the community leaders, and collect a list of the head of household names.

Each of the community leaders will assign a knowledgeable community resident to escort the census team member to the households under his/her jurisdiction. The household will be assigned a unique identification number which will be affixed to the door. These numbers will correspond to a community number followed by a unique three digit ID for that household. At the end of the census, the census team leader will go through the community to be sure that there is no door that is missing a number. If a household prefers not to have a permanent number affixed to the door, they will be asked to at least have a plastic sheet with the number taped to the door for the duration of the census so that they are not visited and enumerated more than once.

For each household, the census team member will ask to speak with either the male or female head of household to obtain a list of the names of all persons resident in the household, ages and gender. The definition of resident in the household means a person who has slept in this household for at least three month in the past or who intends to reside with the family for the next six months. The age of the children needs to be as precise as possible, as they comprise our sample. Event calendars specific to each location will be used to tie ages to such events as elections, weather events, significant deaths, etc.

Other demographic information to be collected in all sites will include education completed by the head of the household, distance to the closest source of water, and observations on clean face status of children, presence of latrines, and cleanliness of the area around the doorway of the household. These measures enable us to further characterize households in communities where

sustainable reduction in disease and infection has occurred, and enable us to measure any environmental effect on reduction in infection.

The census data are entered into the data base using customized data entry forms in access that are standard across all clinical sites.

5.3 Interim Census

The census will be updated at each year for the following three years. The procedures are exactly the same for new households that come into the village since the previous census. The same census information will be collected on the household and each person in the household. For new additions to the households, or loss of persons in a household, addendums to the census list will be made indicating the age, gender, and relationship to head of household for the additions, or reasons for deletion of a person (death or permanently moved to another community). If the person has simply moved within the village, they will be deleted from their previous household and added to the new household using the update census form.

These updated census lists will be used to draw a new random sample of children at each yearly survey, and provide the denominator for the mass treatment interventions.

Census data will also be updated during the time of the surveys. If children have died or moved, or households have moved away, then census update forms will be generated at the time of the survey to update the census list. The data from the surveys will only be able to update the status of children who were randomly selected for survey, but we will capture that information.

Chapter

6

Chapter 6: Procedures: Study Visits

6.1 Overview

The purpose of the study visits is to collect the basic data on the sentinel children who will represent the community burden of trachoma and ocular *C. trachomatis* infection over time. Once the sentinel children are selected in each community, the study team will examine them for trachoma and take swabs for determination of laboratory evidence of infection. Swabs are sent to the laboratories for processing. New cross-sectional samples will be drawn at each study visit, using the latest census survey as the sampling frame, and following the random selection procedures. The sample of 100 children can be examined in a single day, so we have set aside one day for the examinations and a second day for any wrap up. For logistical reasons, the teams cannot make more than two visits to a single community, so children who are not available for the examination within two days will be replaced from the alternate list.

After the baseline visit, the villages receive mass treatment according to randomization schedule with follow-up visits and further mass treatments scheduled thereafter for three years. In the next sections we describe the content and procedures for the baseline and follow-up visits.

The study team for these visits consists of an intake clerk, a trachoma grader/photographer/lab technician, and a driver. There maybe separate persons who perform the functions of trachoma grader, photographer, and lab technician, and person who may perform tubing. A person whose job it is to solely flip lids may also be part of the team, and can help the others be more efficient. Because these functions may be combined in different ways, we describe the job, rather than the person.

6.2 Baseline Visit

Once the child is selected, the family is notified of the selection. In some cases the child is examined in his or her home, and in other cases the family is asked to bring the child to a central location in the neighborhood.

The child arrives for the examination and is checked in by the intake clerk. The intake clerk has a Master List of eligible children for the community and a stack of pre-printed ocular examination forms with the child's study identification information on the form. The intake clerk checks off the child as arriving on the Master List. The intake clerk completes the informed consent procedures. If the guardian agrees to have the child be part of the survey, then the intake clerk signs

the witness for verbal consent form. The intake clerk hands the guardian the correct ocular form with the attached labels, and instructs the guardian and child to wait for the examination.

The intake clerk keeps count of the children who are examined, and notes those who have failed to show up for an appointment. S/he assigns the community health worker to go to the households of children who have not appeared and determine the reason. We will make every effort to include such children, including offering transportation to and from the exam site, or a home visit. If the child has moved permanently or died, then the intake clerk will select a household and child from the alternate list, and complete a census update form. If the child is not available during the examination times in the community due to travel or illness, the reason is noted on the Master list, and again an alternate child is selected.

At the end of the day, the intake clerk must account for all children on the Master list of eligible children. Either they have presented for examination, or a reason is provided for non-attendance, and an alternative appointment scheduled or an alternate selected. If the list is incomplete, the survey team must wait until the households are visited and the child is examined, another appointment made, or an alternate is examined.

The following describes the details of the procedures for the examination. An ocular exam form is completed as a record of the examination.

6.2.1 Trachoma Assessment

Equipment needed: ocular exam form, black pen, torch light, 2.5X loupes.

Before examining the child, the grader should examine the ocular form, be certain it contains labels for the swab and check if a photograph is necessary. The examiner should call out the child's name and ascertain that the child (by name age and gender) is correct. If incorrect, the child and guardian should be sent back to the intake clerk for resolution-either the form sent in was incorrect, or the wrong child was brought for the examination.

To examine the patient for trachoma, it may be necessary to have guardian hold the child securely in the lap, with the child's legs around the guardian's waist and the guardian holding the child's hands. The child's head is then in the lap of the examiner/lid flipper. The child is told to look "down" at the guardian, as it is easier to flip lids. Once the person who flips the lids has on a new pair of gloves, s/he should NOT touch the child anywhere else but on the eyelid to ensure no contamination. Trachoma is assessed by flipping the upper eye lid and examining the tarsal plate for evidence of trachoma. Be certain that the pressure on the upper eye lid as it is everted is not too strong or it will blanche and make grading very difficult. If necessary, let go of the lid between grading and photography. The left eye is flipped first, and graded, followed by the right eye; the right eye is then photographed (if selected) and swabbed. The person who flips the lids will be wearing a pair of gloves which are changed before each new patient. The trachoma grader or lab technician may or may not be the "lid flipper".

We will use the WHO simplified grading scheme for trachoma, which assesses the presence or absence of follicular trachoma (TF), intense trachoma (TI), conjunctival scarring (TS), trichiasis

(TT), and corneal opacity (CO).[6] For this study, the relevant signs are TF and TI, signs of active trachoma. TF is defined as five or more follicles in the region of interest of size 0.5 mm. If there are fewer follicles, then TF is graded as not present. TI is defined as inflammation severe enough to obscure 50% of the deep tarsal vessels. Occasionally, but rarely in children, there is scarring sufficient to obscure the vessels-however, this is not TI as the obscuration is not the result of inflammation. Where scarring is this bad, the eyelid may be labeled as "cannot grade" for signs of active trachoma. The data on the presence or absence of trachoma signs is entered onto the form.

If more than one grader is used, graders will be assigned randomly to intervention and control communities, to ensure no subtle grader bias due to doing only one kind of village exists in the treatment arms.

6.2.2 Laboratory Procedures

6.2.2.1 SPECIMEN COLLECTION

Equipment needed: sterile swabs, swab vials, labels, ocular swab shipping list.

Each sentinel child must have an ocular swab taken for determination of infection. If the child refuses the swab, or a swab cannot be taken for another reason, then the child is ineligible. The intake clerk should be notified that the child is ineligible, a note made in ink on the ocular examination form and in the Master List of Eligible Children, and an alternate household and child selected as replacement.

Each ocular form has a set of printed labels with the child's study identification number. One label is placed on the ocular form in the section asking if a specimen was taken, one on the study vial after the swab is inserted, and one on the shipping log for the box in which the vial is inserted. In this way, each vial box has an accounting of specimens for subsequent shipping. There are extra labels as well, if one is lost or torn.

We will be taking a field control swab for each laboratory technician sufficient to have 50 control swabs per survey. These will be taken using the "blue air" swabs that will be pre-marked in each box of swabs.

6.2.2.2 PROTOCOL FOR SWABBING OF THE CONJUNCTIVA

1. Upon taking a swab from the box, the lab technician will announce what type of swab it is:
   - An unmarked "plain swab", indicating no control collection necessary for this patient.
   - A "blue air swab" (negative field control), indicating examiner must collect a second swab for this patient. Swab sachet will be marked with blue color.
2. The swab sachet should be opened in a sterile manner, revealing only the tip of the swab shaft, with the swab head itself remaining sterile deep within the sachet.

3. If the child needs an ocular photograph, this must be taken prior to the swab. When ready to swab, the lab technician should slowly pull out the swab (if removed too quickly the Dacron swab tip can unravel). The gloved hand should be held no closer

[6] Thylefors B, Dawson CR, Jones BR, West SK, Taylor HR. A simple system for the assessment of trachoma and its complications. *Bull World Health Organ*. 1987;65(4):477-83.

than 1 inch from the Dacron polyester swab tip during the entire swabbing procedure, in order to avoid contamination of the swab tip.

4. As the lid flipper (who may be the technician) holds the right eyelid in the everted position, the technician swabs the upper tarsal conjunctiva of the child with a gloved hand, using a steady and firm swab. The swab should be placed flat, with its entire length parallel to the conjunctiva to give the greatest surface area of contact, and if necessary, this may require repositioning of the child's head by the examiner. The swab should be drawn firmly in one direction over the conjunctiva with enough pressure to cause blanching of the conjunctival vessels. The swab should then be rotated 120 degrees along its axis and the newly revealed fresh region of Dacron on the swab tip should now be drawn firmly across the conjunctiva. The swab should be rotated another 120 degrees along its axis and the conjunctiva swabbed for a third time. This will ensure sufficient collection of conjunctival epithelial specimen for PCR analysis in the lab.

6.2.2.3 PCR CONTROL SWABS

A negative field control ("blue air swab") will be taken on a randomly chosen 5 children per village to assess the frequency of contamination. For each negative field control, the examiner will pass a sterile Dacron swab within 1 inch of the child's conjunctiva. Control swabs are taken after the original swab but before changing gloves for the next participant. A label from the control set, made to look like a regular ID but indicating a control swab will be used. One label is affixed to the ocular form in the indicated area and one is affixed to the vial.

6.2.2.4 PROTOCOL FOR TUBING AND HANDLING OF SAMPLES

The tubing and handling protocol must be carefully followed in order to prevent contamination and ensure the safe transport of the samples to the microbiology laboratory. The person in charge of labeling, tubing, arranging, and handling the samples needs to perform this task in the most orderly and attentive manner.

1. The tuber should wear gloves on both hands at all times. The tuber's gloves only need to be changed when any potential contamination of the gloves occurs. The tuber opens the capped, hinged lid of a microcentrifuge tube, which has been labeled with the participant's random identification number. If it is a control or "blue air" swab, then the label comes from the control set of labels.

2. The swab is inserted by the lab technician (using the still-gloved hand that swabbed the participant's conjunctiva) into the microcentrifuge tube held by the tuber. The swab shaft should only be inserted until the Dacron swab head is fully in the tube. The tuber should lower the cap onto the swab shaft held by the examiner, and the examiner should quickly break the swab shaft using a swift downward snapping wrist movement.

3. The tuber will screw the cap of the microcentrifuge tube tightly and place it in the sample collection box, located in the cooler bag filled with frozen ice packs. The flap of the cooler bag should be closed between each patient. The cooler bag should be in as cool a place as possible in the field, in a shaded area out of the sun.

4. Upon returning from the field each day, the samples will be immediately taken to the health center and stored in a freezer, reserved solely for storage of specimens and ice packs.

All samples will be in sample boxes, labeled with the village and district names for easy future identification.

6.2.3 Shipping and Storage of Samples

In accordance with the Roche COBAS AMPLICOR™ CT/NG protocol, swab samples taken in the field will be transported on ice in a closed, insulated container until arrival at the Proctor Foundation Microbiology laboratory in San Francisco. Samples will be imported to the USA per the CDC Permit to Import/Transfer Etiological Agents or Vectors of Human Disease.

1. Materials for shipping to the US
   - Insulated shipper box (ThermoSafe VIP insulated shipper, 615DCS or 499 DCS)
   - Gel Packs (ThermoSafe U-tek 24 oz. -10°F)
   - Shipper label
   - Consignee label
   - Responsible person label
   - UN3373 label
   - Biological substance, Category B label
   - Documentation to include in shipper box
     - Pro-forma invoice
     - Letter from Shipper
     - CDC permit
     - Letter of approval from Ethics committee in country
     - airway bill from ;world Courier, DHL, or other air transport service

2. Packing the samples

For dry samples without media:

- Boxes can be wrapped with plastic wrap and clear plastic tape.
- Boxes may also be wrapped with a cotton absorbent pad (chuck) for improved insulation.
- As soon as samples are prepared for shipping, they should be returned to the -20°C freezer to prevent samples from thawing.
- Place all stickers on outside of the shipper box (see detailed shipper instructions).
- Leave at least one shipping box, preferably a partially-filled one, unpacked for inspection by the air shipper employee and or customs.

When ready to ship, the prepared sample boxes should be placed into the partially filled shipper box.

Next, all frozen icepacks and samples should be placed in the shipper box. (6 gel packs for a small shipper, 12 gel packs for a large shipper)

- For a small shipper box: Use 6 frozen gel packs-- one below sample boxes, 4 on each side, and one on top of sample boxes
- For a large shipper box: Use 12 frozen gel packs-- 6 gel packs flat at the bottom and 6 on the top

Immediately after the shipment is accepted by the shipping company, the shipper box should be closed and taped shut using clear plastic tape. Tape should be placed around all corners of the outside box.

6.2.4 Laboratory Processing Procedures

A dry swab is taken in the field, a study identification label is affixed, and the vial is kept frozen until it reaches each country team's designated laboratory as described above. The specimens are processed according to strict protocol, outlined in the manufacturer's kit directions, followed by the Microbiology Laboratory at the Proctor Foundation. We will use the Roche Amplicor *C. trachomatis* qualitative PCR assay from Roche Molecular Systems. Procedures are summarized as follows: Each swab is eluted by vortexing in Amplicor CT/NG lysis buffer in polypropylene tubes, then Amplicor specimen diluent is added. Using a known positive sample in the laboratory, we will create positive and negative C. trachomatis (CT) processing controls; two CT+ and two CT- processing controls are run with each batch of specimens. The Working master mix is created and the specimens prepared prior to amplification. The specimens and controls are placed in the thermal cycler for amplification. Once completed, the specimens are denatured, and sent for detection using probe-coated microwell plates. Detection is accomplished by measuring the optical density at A_{450}. The assay result for the negative controls should be less than 0.2 A_{450}, and the assay result for the positive controls should be 0.8 or greater for ocular specimens for a valid run. We will test separately for amplification of both the target plasmid DNA and the master-mix internal control samples to determine if PCR was inhibited. Samples whose values in valid runs are ≥0.8 A_{450} are counted as positive, and samples less than 0.2 A_{450} are negative. Samples for which the result are equivocal (≥0,2, <0.8) will be tested again; if equivocal twice, they are left as equivocal and called not positive in the analyses as no run equaled 0.8 or greater.

6.2.5 Photograph Grading

Equipment needed: camera, memory card, labels, fully charged battery, photolog book, CD burner

Optional: extra battery, memory card reader

An ocular photograph will be taken of the right eye of every nth child sufficient to ensure at least 50 photographs per trachoma grader per visit.

Clinical photography will be performed **before** conjunctival swabbing. A handheld Nikon D-series camera with a 105mm f/2.8D AF Macro Nikkor Autofocus Lens (fully extended, in manual setting) will be used. If possible, use natural light to help position the patient but all photos are taken with falsh engaged. Camera set-up is as follows:

- Image settting .jpq, fine, large
- White balance A or Flash
- Flash from camera body snapped up into position.
- Aperture-preferred, set at F32 or higher
- ISO 320- this can be increased if needed to maintain high F-stop

Several photos will be taken: first, a photo will be taken of the patient identification number (which can probably be done with or without flash). The image is brought into focus by coming to the appropriate distance from the conjunctiva. Note that the camera itself is not focused—the

macro lens should always remain in the fully extended, 1:1, manual setting. A sharp image is obtained by positioning the camera and lens, not by changing the focus of the lens. At least two photos will be taken of the right upper conjunctiva. The patient is placed into the examination position, either sitting, standing, or in the "head-clamp" position. The photographer focuses on the blood vessels of the upper lid, getting them (if visible) into sharp focus. If the eyelid is blanched from being everted, pressure from the everting finger can be reduced. Two photographs of the eyelid are taken.

Photos are immediately reviewed for grade-ability and re-taken if necessary. All of the following are considered unacceptable photos (with possible solutions in parentheses):

1) out of focus (OK to increase F-stop higher than F-32 to increase depth of field).
2) too much water on the conjunctiva (can gently dab with swab—don't rotate or move swab across conjunctiva, as this can create inflammation)
3) not centered (hold child more firmly, photographer can rest arm to steady)
4) conjunctiva not fully exposed (OK to use more than one finger, hand, or swab for better exposure).
5) Conjunctiva blanched from too much pressure everting (reduce pressure, allowing blood flow back into conjunctiva.

If the child cannot be photographed for some reason, it does not affect the eligibility of the child. A notation is made on the ocular form of why the photograph cannot be taken, but no replacement is sought. Again, this is expected to be a rare event.

Images are stored in digital file folders according to study ID number. They are burned onto a CD every week and sent to LSHTM where they are graded for quality assurance purposes by Dr Robin Bailey, who is the standard grader for the PRET study.

6.2.6 Follow-up of Children

All children on the Master List of Eligible children for each community will be followed up with if they are not initially available for examination. If necessary, families will be offered free transportation to the examination site and back, or a home visit in order to assist in overcoming barriers to the examination. If the child/household is temporarily away, another appointment will be set up for a second day. If the child is too ill to be examined, refuses, has died, the household has moved permanently, or travel exceeds the time the study team can be in the community, then the next household and child from the alternate list will be selected to participate. When the alternate child is selected, the Master list will be updated to indicate which child is being replaced, and the Study Identification number of the replacement child if additional data on the child or household is found that necessitates a change to the census list, then an update census form will be completed.

For the longitudinal study villages where all children under age 10 years are enrolled, efforts will be made to include all children as there are no "replacements."

6.3 Forms and Swab Management for Visits

At the end of the examination day, when all children on the Master List of eligible children and the alternates have been accounted for, three reconciliations will take place. First, the lab technician will be certain that each tube has a label. If one is missing, the forms and tubes should be matched to determine which label is missing. If more than one tube is missing a label, then the children may need to be re-swabbed to be certain the trachoma grade corresponds to the correct

laboratory determination of infection. The number of tubes must also match the number of ocular examination forms. If there is a mismatch, then the tubes and forms should be matched to determine what is missing. If an ocular examination form was missing or not filled in, the child must be re-examined. Similarly, if a tube is missing, and there is no notation that a specimen was not taken for a reason, then the child must be re-swabbed. Once the swabs and ocular forms are reconciled, the ocular forms are given to the photographer.

The photographer reviews his/her photolog to be certain that where a child was to have digital images of the eyelid that the photograph was taken or a notation made as to why the photograph was not taken.

The intake clerk will compare the ocular exams in hand to the Master List of eligible children to be certain that each child who presented was examined. If an ocular form is missing but the child is listed as having presented, then the study team must return to the household and reconcile the difference, likely by re-examining the child.

The intake clerk must account for each child on the Master List of Eligible children for the community, either with an ocular form, a notation on the list (in which case there is an alternate child chosen); also if the notation states the child has died or permanently moved out of the community, or moved within households, then a census update form should also be present.

6.4 Missed Visits

Missed visits, for the purpose of this trial, occur if a community cannot be seen within the window of plus or minus two months. The four month window keeps the village visit largely during the same season as the original scheduled visit.

6.5 Follow-Up Visits

The first mass treatment is carried out immediately after the baseline survey. Subsequent follow-up visits are at 6, 12, 18, 24, 30, and 36 months from the first mass treatment. The census lists are updated formally every year prior to the 12, 24, or 36 month survey. The 6, 18, and 30 month surveys will use the census lists for the previous survey when drawing new random samples of sentinel children. The window for each survey is +/- two months.

The exact same procedures as stated for the baseline examination visit are to be followed for all subsequent examination visits. In all cases, mass treatment according to treatment arm is to follow immediately after the survey, to ensure that children are not treated prior to the survey.

6.6 Study Visit Sample Schedule

As noted earlier, we have two days in each community to schedule examinations. Depending on the starting month, we have flexibility in the schedule to work around holidays and rainy seasons. If the rains are heavy, we will alter the schedule of surveys, and allow a window of +/- two months for the community examinations.

Chapter

7

Chapter 7: Intervention: Mass Treatment Implementation

7.1 Overview: Treatment with Azithromycin

The intervention for this trial is community treatment with azithromycin, a single dose antibiotic highly effective against *Chlamydia trachomatis*. Antibiotic sufficient for this trial has been donated by Pfizer international to the National Trachoma Control program in Niger. The dosage is 20mg/kg, up to 1 gm in a single dose of either liquid (for children) or tablets (for children able to swallow tablets and adults). Children below 6 months of age are not treated with azithromycin but are given topical tetracycline for trachoma.

Azithromycin has been extensively studied for mass use in trachoma control, and has been found to be very effective and well–tolerated. In the ACT trial, no significant adverse effects in children and adults were noted in Tanzania, Egypt, and The Gambia[7]. In the STAR trial, which treated adults, no differences in adverse events were noted between topical tetracycline and oral azithromycin[8]. Initial potential concerns for creating clinically significant resistance in other organisms to azithromycin do not appear to be realized[9]. On the basis of these and other studies, Niger has undertaken mass drug administration for their communities, with education to residents that any GI concerns be directed to local health centers and posts for treatment. We anticipate using the same local network to monitor any self reported adverse events.

7.2 Six monthly Mass Treatment

Models imply that children form a core group for the transmission of trachoma—if infection can be eliminated in this age-group then it would presumably fade away in adults, whose treatment now requires a substantial portion of a program's resources. We have demonstrated that treating children alone can be successful in an area with a modest amount of trachoma. A recent study in a

[7] Schachter J, West SK, mabey D, et al. Azithromycin for Trachoma Control. Lancet 1999;354:630-5

[8] West SK, West ES, Alemayehu W, Melese M, Munoz B, Imeru A, Worku A, Gaydos C, Meinert CL, Quinn T. Single-dose Azithromycin prevents trichiasis

[9] Gaynor B, Holbrook KA, Witcher JP, et al. Community Treatment with Azithromycin for trachoma is not associated with antibiotic resistant S. Pneumoniae at One year. Br J Ophth. 2003;87:147-8

hyperendemic area of Ethiopia also demonstrates a degree of community protection in older, untreated individuals after quarterly treatment of children only.

Therefore, in addition to evaluating two coverage targets for the entire community, we will be evaluating whether biannual treatment of children is as effective as annual treatment of all individuals in a community. 12 villages will be randomly assigned to a coverage level of 80%, and 12 to 90%, with children ages 0-12 (i.e. newborns to those less than 13) receiving treatment every 6 months.

Bi-annual treatment visits will be organized and implemented in the same way as annual treatment visits, except that only children 0-12 will be treated in these communities (12 villages assigned to standard coverage and 12 assigned to enhanced coverage). Any questions regarding this exclusion category by community members will be clearly addressed and explained by local health workers, who will have received training in this area. All children between the ages of 0-12 who present for treatment and have guardians present for consent will be treated.

7.3 Monitoring Mass Treatment Coverage

The census data that are obtained and updated yearly will be the primary tool used for monitoring mass treatment coverage. The programs that are built for data entry of the census data will have a report that will be generated, consisting of mass treatment lists for each community. These master lists are used to record observed treatment coverage for each resident of the community. The data entry of these lists is essential to monitoring coverage as the treatment is underway.

After each community has completed its mass treatment, the treatment data set is sent to the principal investigator within a week in order to monitor the success of the treatment team in reaching coverage targets. S/he will review the percent coverage of the community, and if it is over or under target, the reasons why in the file. This will be done within three weeks of mass treatment ending so that if necessary, the treatment team can be re-deployed to the community to improve coverage. If all residents are accounted for, and the resident did not meet treatment coverage targets, the principal investigator will discuss with the treatment team supervisor ways to improve coverage for the second or third rounds (perhaps different timing or working with other community health team members, for example).

7.3.1 Mass Treatment Verification Procedure

Treatment verification is critical to ensuring that coverage levels have been achieved and that reported data is accurate. Treatment verification occurs after each community has completed mass treatment and is conducted by the Community Drug Distributers (CDDs) who initially treated the villages. Post-mass treatment, a CDD pair, consisting of a man and a woman for each sub-village, returns to the village to randomly select a sample of no less than five houses for interview. During the interview, the CDD pair records the number of people residing in the house and how many of those people received treatment on a treatment spot-check form. After the sample of houses is interviewed, the CDD pair compares the information recorded on the spot-check form to the data in the census treatment books as well as the information gathered from the household's respondent during team members initial visits to the home prior to mass treatment.

7.4 Masking Intervention

These trials are single masked, as it is not possible to mask the communities to the assignments they are to receive. There are two outcomes-clinical trachoma and infection-based on data collected by the survey teams. The teams that implement mass treatment are different than the teams that will be undertaking the survey. The survey team will be unaware of the assignment to coverage arm (80% versus 90%), and this element of the trial will be masked to the trachoma graders. For villages randomized to target children for treatment, the survey teams are liable to be informed of treatment status by families.

Chapter

8

Chapter 8: Cost Effectiveness Data Collection

8.1 Overview

The cost effectiveness analysis will be based on community cost estimates that we will calculate using cost collection instruments. For each community, we will collect cost information regarding: all medical and non-medical personnel involved in the study and all consumables used. We will also include laboratory costs attributable to screening/treating each community. The totality of the equipment costs - such as vehicles and field equipment costs – and laboratory costs will be recorded aggregately and then allocated to each community according to the community population.

For each intervention, a designated member of the team will fill out the recurrent costs forms. Equally, this designated member of the team will be in charge of filling out the capital costs that are only incurred once for resources shared across different communities.

The filled forms will be double entered using the data entry software "Access" by two different data entry clerks. The double-entered data will be verified, and any differences in data entry will be checked against the paper forms.

8.2 Cost Data Collection

Cost data to be collected are of two types: 1.Recurrent costs and 2.Capital costs (i.e. those only incurred once). The first kind of data will be recorded each day in the field. The second type of cost data will be recorded aggregately for the study and allocated to each community according to its relative population.

The cost data collection will be done by filling out the following five forms.

- Form 0: Capital Costs
- Form 1: Medical Personnel Costs
- Form 2: Non-Medical Personnel Costs
- Form 3: Field Consumables' Costs
- Form 4: Lab Costs

Chapter

9

Chapter 9: Study Instruments

9.1 Overview

The data to be collected for this study consists of household information, clinical, and laboratory information for each child. In addition, data are collected on the mass treatment intervention and details of the study process. In the next section, we describe the forms that are used to collect these data.

9.2 Census Forms

The baseline census form is used to collect information on each household in the communities randomized to this trial. It is the central document that provides information for the following:

- Selection of households with eligible children in the community
- Selection of children within a household when there is more than one eligible child
- Master List for Surveys
- Data base for mass treatment
- Environmental factors at the household level that may be important for trachoma

A household (concession/compound in Niger) is a family unit sleeping regularly under the same roof. The definition of resident in the household means a person who has slept in this household for at least three months in the past (or if age less than 3 months, was born into the household) or who intends to reside with the family for the next six months. In this section, details are provided on filling in the Baseline and Update Census forms.

9.2.1 Baseline Census Form

Identification numbers:

VILLAGE: __ ___

In Niger, use the first numeric two digit entry code to identify the village.

HOUSEHOLD NUMBER: ___ ___ ___

Use this three digit alphanumeric entry to uniquely identify a household; this number is also written on the door of the household.

Village Name: write in the name of the village

Name of head of Household: Write in the name of the head of the household. This may be provided by the neighborhood leader or another neighbor if the head or another adult in the family is not home.

Census taker: insert the initials of the census taker for this household. Each census taker should have a unique set of initials, including a number if there is more than one person with the same initials.

Date: insert the date in which the household was completely censused (or the date at which census gathering had to be stopped and no information was provided). Use DD/MM/YY format.

1. Is there an adult home to answer questions?

If yes, mark "yes". If not, the census taker should determine when would be a better time to return to get the information. If the family is traveling and not returning during the time of the census, then the census taker must indicate the reason for being unable to census. If there is not information on the family, s/he MUST return at least three times at different times over the course of the village census to try and catch an adult at home. If the census taker is unsuccessful, then mark "no" unable to census and provide a reason why. This household will not be chosen as a source of sentinel children, as detailed information will not be available on the children, but census information may be provided at the time of mass treatment, if the family returns for that activity.

2. How many years of education has the head of household completed?

Ask the question of the adult, and make sure that the answer is years completed, not including starting but not completed. Write in the number of years. If the answer is a grade, translate that into years completed. If the adult does not know, write letter "X" for unknown.

3. How far away is the nearest water source?

We will measure the distance to water by asking how long it takes to walk one way directly to the nearest source of water. Time will be measured relative to the time it takes to do common tasks, such as boil water, cook rice, or other tasks. Once the persons can provide an estimate, code as less than 30 minutes, 30 minutes to one hour, or more than one hour.

4. Has there been a health education program to promote face washing in this village in the last year? I do not mean a radio campaign but a local program in your village.

This is an optional question for areas in which the full SAFE strategy has been implemented. It helps determine if the health education campaigns have reached the village level.

5. OBSERVE: Does this household have a latrine:

The census taker will look in the compound or area around the house for a latrine. If one is not visible, the census taker will ask the interviewee if the household has a latrine. If yes, the team member will ask to see it. If it is within 60 feet (20m) of the house, it will be counted as a latrine for that household.

6. OBSERVE: Is the doorway area clear of garbage or waste?
The census taker observes the cleanliness around the doorway of the house. S/he will be standardized to observe the area around the doorway for evidence on the ground of leftover food or animal or human waste. Waste water or plant detritus does not count as waste. If there is a large animal pen within 25 feet (about 8 m) of the doorway, this will be recorded and counted as unclean doorway.

7. Household List: note that to the left of each name slot is a pre-printed number in sequential order. This two digit code is the unique person code for each person in the household. Use it when needed to identify the mothers of children as described below.

Name: List the names of all persons who reside in this household, as defined above. Start with the head of household. List the first name, last name, and an optional place for alias if needed.

Sex: Note the sex of the person as "M" for male and "F" for female.

Date of Birth: If it is possible to obtain a date of birth, record the date of birth: use DD/MM/YY format. If only the year is known, fill in "x" for the unknown numbers. Use a vaccination card or MCH card if available to get the birthdate of children, and a voting card or other piece of ID for the adults if available. If the entire date is unknown, then skip to the column labeled "age."

Age: use this column if no birthdate, or at least year of birth, can be obtained. Use an events calendar specific for each region to estimate the age of the person. If necessary, have other family members assist in determining the age. Avoid having a consensus using round numbers or last digits with 5's as it indicates digit preference; try to get the person and/or family members to b e as precise as possible. For children age less than ten, indicate years and months if possible. For children less than age one year, insert a zero for years.

Mom: For each child age less than ten years, insert the two digit person code for the primary caretaker of the child. This will help identify the child in subsequent surveys.

Child Face status: The census taker will make observations on the cleanliness status of each child ages five years and under who are present at the time of the census. S/he will be standardized to measure a clean face as the absence of ocular discharge or "sleep" on the eyelashes or lids, the absence of nasal discharge on the nostrils, cheeks, and lips, and the absence of flies on the face when observed for 3 seconds. The presence of any one sign is evidence of an unclean face. If the child is not present or cannot be observed, indicate "X" for each sign as "don't know". Otherwise, record yes or no for presence of sign on the child's face.

Random number: an optional column for study sites where the data entry cannot be completed prior to selecting an eligible child in a household, and a random number assignment is made in the field. In the field, the census takers will have a Table of random numbers with a random

start. They will then go horizontally across the page as they assign each child, starting with the first closest to the top, a random number in order that they appear on the table. As the number is assigned, it is crossed off the table. The child with the lowest number is the one selected from that household. For example, if child 04 has number 54 and child 05 has number 16, child 05 is the selected child. Note that if child 05 is not available for the survey, another household entirely is selected, not another child from the same household. This avoids always selecting younger children as they tend to be around the house.

9.2.2 Census Update Forms

9.2.2.1: SURVEYS AND MASS TREATMENT UPDATES (FORM 2)

After the baseline census, surveys will be undertaken and mass treatment provided. Each of these contacts provides an opportunity to update the census information for the village. The Master List of eligible children for the survey has a place to note if the child has died or moved permanently from the village. If these are the reasons the child was not examined, then a census update form is generated for data entry. Similarly, if a resident has been added to a household at the time of mass treatment (who is not a visitor), or a resident has died or moved, this information is noted in the mass treatment book and generates a census update form. The Census update asks for the complete identifying number of the person. This is the village number, household number and person number. Assign the next available number in the household to the person to be added.

If the person is to be added: fill in the information on the new addition exactly as described for the baseline census:

Name, Sex, Date of Birth, Age, and Mom are filled out the same as described in Form 1. Note for new additions if they are children, the face status is not needed.

If subtracting a person from a house: check that the person whose ID is listed is to be subtracted from the household and provide a reason. If the person is known to have moved to another household in the village, check #3, and provide the household number where the person has moved. If the household number is unknown, provide the name of the head of the household and as soon as possible, locate the household number for that head of household. Generate another census update form to add those persons to their new household. If the reason for subtraction is not death or re-location, check “other” and specify the reason. If the person is in the village but refused to be surveyed or mass treated, DO NOT fill in a census update form. This is only to be used if the location has changed.

If subtracting an entire household from the census, you do not need to insert a person number, but item #3 on change code must be checked. Provide a reason for the subtraction. The result will be the elimination of that household number, so if the entire household has become part of another household (reason #2) then be sure and have the number of the other household, and add the persons to the household with another census update form.

9.2.2.2 YEARLY CENSUS UPDATES (FORM 3)

The census will be updated every year in preparation for drawing a new sample, and undertaking mass treatment. A Master Census list of each house will be printed, using the most

recent census update data, likely from the six month survey previous. The census takers will use this book to return to each house and update the census.

If adding new persons, use the same information as in the baseline census form:

Name, Sex, Date of Birth, Age, and Mom are filled out the same as described in Form 1.

If subtracting a person, indicate the reason as described above: If the person is known to have moved to another household in the village, check #3, and provide the household number where the person has moved. If the household number is unknown, provide the name of the head of the household and as soon as possible, locate the household number for that head of household. Generate another census update form to add those persons to their new household. If the reason for subtraction is not death or re-location, check "other" and specify the reason.

If the entire household has departed the village, then note the household has left permanently. There should be tangible evidence of permanent re-location and not just traveling. Temporary absences of households of less than one year should not be changed in the census unless the neighbors/village leaders know they have gone for good.

In the event that a new household has come into the village, then the census taker should assign a new household ID (use the next available household number on the village list being certain that it has not been assigned elsewhere). Add the number to the doorway. Fill in a census form identical to the baseline census form with household information noted as described above.

9.2.3 Master List of Eligible Children for Survey

As the examination surveys are undertaken, the intake clerk will have a master list of children who have been randomly selected for the surveys (Form 4). On the list is a column that allows reasons to be given for why children are not examined. If a child is not examined due to death or a permanent move out of the village, then a census update form is generated by the intake clerk, and that form is entered into the census data base and updates the census files.

9.3 Ocular Form

The top of the ocular form (Form 5) is pre-printed with information from the census data base. There should also be affixed a set of four labels. These labels are used for the specimen vial, the specimen log book, and the photolog book if necessary and the field control swab if necessary. The examiner should observe the form for a star in the upper right corner, which indicates the child needs an ocular photograph of the right eye.

Child Study ID number: the nine digit code consists of a two digit village code, followed by the household number followed by the person code.

Examiner: the person who will be doing the trachoma grading should insert initials. These initials will be checked against a data base of certified graders.

Date: the date the exam was performed should be written in. The format is DD/MM/YYYY.

Child's name, Age, and Sex: as it appears in the census database will be printed here.

Head of Household name: the name of the head of the household where the child resides is listed here as it appears in the census data base.

(NOTE: The age and sex help identify the child that should be examined, in case there is any confusion. The head of the household name is useful to locate the child or perform a house visit if necessary)

1. Was the ocular exam performed on the child?
   The appropriate box is checked. If the child was present but no exam was done, a reason should be specified. If answers 2-5 are checked, then the first child off the alternate list should be informed and brought in for examination. If answers 4 or 5 were checked, then a census update form also needs to be filled out to ensure the census data base is updated.

2. Classification of trachoma
   The examiner first examines the LEFT EYE. S/he grades the eye for trichiasis and corneal opacity. The eyelid is then flipped and signs of TF, TI, and TS are graded as present: "1" or absent: "0". If they cannot be graded at all, use grade "9" for cannot grade.
   The examiner then examines the RIGHT eye. The grading is repeated. The examiner inserts "0" or "1" as necessary. No space should be left blank-if the sign is not present, a "0" must be written to be certain the sign was not skipped.

3. Was a swab taken?
   A swab must be taken of the eyelid of the right eye (if the child is selected for photography, then the image is taken prior to swabbing). If yes, then the examiner must write in the box number in which the vial is placed, and place an ID label next to the box number. The label helps to make certain the vials are not mixed up. If the swab is not taken a reason must be provided, and a child from the alternate list is informed and brought in for examination.

4. Was this child chosen for photography?
   If yes, then tick off the yes box and place the ID label next to the yes box and another label in the photography log book. The first image taken is of this identification number in the photography log book.

5. Was this child chosen for a field control lab swab?
   If a blue colored swab was selected for this child, then the child is to have an air swab taken. In this case, a special set of field labels have been created which resemble regular identification numbers but are laboratory controls. Place one of these triplicate labels next to the yes box, to link the child to the field control; place the second label on the vial, and place the third on the specimen shipping list for the box in which it will be inserted.

9.4 Photolog Book

The purpose of the photolog book (Form 6) is to keep a record of the photograph images taken as back up in case there is an error in the order the images are taken, or the ID photo is missing. The photolog book consists of sequentially numbered columns. For each person to be

photographed, the ID label with the form is placed in the next available slot, and the image is taken of that identification number. This link enables the clinical grade to be associated with the photograph grade. Then the photographer takes two images of the upper eye lid. S/he reviews the images, and if they are acceptable, crosses off the next two slots after the ID label in the photolog. If one or both is not acceptable, then one or more are deleted and new images taken until there are at least two that are acceptable for transmittal. There should be an "x" for as many images as are going to be transmitted.

At the end of the survey, the photographer burns the 150 plus images (50 children times a minimum of three images) onto a CD and sends the CD to Dr Bailey at LSHTM, with a copy of the photolog for that survey period.

9.5 Ocular Specimen Shipping List

Each of the specimens taken will be placed in a box for the laboratory that holds 100 tubes. The boxes are numbered sequentially, and indicate the village and date it is filled. As the specimens are taken, a label is placed on the ocular form, on the vial, and on the shipping list. Each page of the shipping list (Form 7) refers to a separate box, so that each page can serve as the list sent with the specimens to the lab to describe the contents of the box. In addition, the box number is indicated on the ocular form, so if a tube is missing, the likely box in which it was packed can be located.

When the page for a box is completed, the page is copied to send with the box when shipped. And the original is kept with the field team as a record of having taken and packed the vial.

9.6 Mass Treatment Log Book

This book (Form 8) is printed out from the census data base at the time of mass treatment, for use by the mass treatment team to determine coverage. Each page is a unique household in the community, organized by village and last name of the head of household. Section I is the listing of the current residents. For each person, indicate with a tic in the appropriate box if they were treated or not, and if not present, why they were not present. If they were treated, then indicate the dose that was provided in terms of number of pills or ml of liquid.

Section II permits adding new residents and indicating if they are temporary residents of the household for the purposes of mass treatment. Sometimes the village is inflated with persons who want treatment, and indicate they are part of a household in case that is a requirement, which in this study it is not for purposes of mass treatment. By adding them and indicating they may be just temporary, it helps account for the doses used but does not artificially inflate the census or coverage figures.

This book is entered into the data base as a record of coverage for the community, and if new persons have come into the house as permanent residents, then the census is also updated.

9.7 Cost

Form 0: Capital Costs

This form contains cost information on any vehicle used during the study and field costs incurred only once. This table will be filled out only once and not for every field trip. Capital costs will be imputed to each village on a population based proportional basis at the end of the study.

The table referring to capital costs for vehicles:

1. Average petrol cost per kilometer/miles: Record the cost of petrol, in local currency, at the beginning and end of the field trial. Add these 2 values together and divide by 2 to get the average cost. Conversions into US$ will be performed at the end of the study.
2. State whether the car was bought or rented
3. State the purchase price, in local currency, when applicable.
4. State the daily cost, in local currency, of the vehicle if it is rented
5. State the years of useful life attributable to the car. This will be calculated based on the year of make of the car and the number of kilometers ran by the car.
6. State the resale value of the vehicle (if it is not resold at the end of the study, state what the likely resale value would be).

The table referring to field equipment capital costs:
For each item of equipment:

1. The number of units
2. The price/unit in local currency
3. State whether the good was donated or purchased
4. If the good is donated, state what its market value would be.

Form 1: Medical Personnel Costs
Form 1 will be filled out for every day in the field.

At the top of the form:

1. The date of fieldwork will be recorded in the format: "1st January 2008."
2. The location(s) of fieldwork (the name of the community/communities worked in)
3. The activity/activities performed that day (e.g. sensitization, census, examination, treatment)
4. State clearly the number of people covered by the activity e.g. "100 children examined", "150 people treated".
5. The distance traveled (state km or miles) that day by each vehicle separately

Medical personnel table:

1. The position of the medical personnel involved (e.g., ophthalmic nurses). Each person is written on a separate row
2. The number of hours spent working that day (including travel to and from location from accommodation)
3. The tasks performed that day by that person
4. The person's normal salary and the over what time unit (e.g. salary/year), in local currency
5. If, and how much (in local currency), the person received as a per diem
6. Any additional costs for that person (e.g. accommodation, food etc.).

7. Any notes that are relevant to understand the extent of the involvement of that person in the study.

Form 2: Non- Medical Personnel Costs
Form 2 will be filled out for every day in the field.

At the top of the form:

1. The date of fieldwork will be recorded in the format: "1st January 2008."
2. The location(s) of fieldwork (the name of the community/communities worked in)
3. The activity/activities performed that day (e.g. sensitization, census, examination, treatment)
4. State clearly the number of people covered by the activity e.g. "100 children examined", "150 people treated."
5. The distance traveled (state km or miles) that day by each vehicle separately

Non-medical personnel table:

1. The position of the non-medical personnel involved (local helpers, drivers, etc). Each person is written on a separate row
2. The number of hours spent working that day (including travel to and from location from accommodation)
3. The tasks performed that day by that person
4. The person's normal salary and the over what time unit (e.g. salary/year), in local currency
5. If , and how much (in local currency), the person received as a per diem
6. Any additional costs for that person (e.g. accommodation, food etc.)
7. Any notes that are relevant to understand the extent of the involvement of that person in the study.

Form 3: Field Consumables Costs
Form 3 will be filled out for every day in the field.

At the top of the form:

1. The date of fieldwork will be recorded in the format: "1st January 2008."
2. The location(s) of fieldwork (the name of the community/communities worked in)
3. The activity/activities performed that day (e.g. sensitization, census, examination, treatment)
4. State clearly the number of people covered by the activity e.g. "100 children examined", "150 people treated"
5. The distance traveled (state km or miles) that day by each vehicle separately

Field consumables table:

1. The name of the consumable (one consumable per row). These items may include: tables, powder, water if bought, dispensing instruments, swabs, gloves, etc. Note that vehicle repairs are recorded here.
2. The cost/unit (in local currency), plus specify what the unit is e.g. "Box of 100 gloves", "Box of 100 swabs"
3. How many units were used

4. State whether the goods were donated or purchased
5. If the good is donated, state what its market value would be
6. Any relevant notes that can clarify the market value attributed to the good when controversial.

Form 4: Lab Costs for PCR
Form 4 will be filled out for every day of PCR lab work.

<u>Capital costs table:</u>

For each item of equipment (e.g. freezer, refrigerator, thermocycler, safety cabinet, pipettes, heating blocks, vortex, tube racks, plate washer, incubator, Microwell Reader, computer, printer):

1. The number of units bought
2. The price/unit in the currency in which they were bought
3. State whether the good was donated or purchased
4. If the good is donated, state what its market value would be

<u>Lab staff costs table:</u>
To be filled in every day:

1. The position of the lab staff member (e.g. lab technician). Each person is written on a separate row
2. The number of hours spent working that day on the study's samples - excluding the lunch break if it lasts more than half an hour
3. The tasks performed that day by that person for PCR of the study's samples
4. The person's normal salary and the over what time unit (e.g. salary/year), in local currency
5. Any additional costs (accommodation, food, per diem) per day
6. Any notes that are relevant to understand the extent of the involvement of that person in the study.

<u>Lab consumables costs table:</u>

1. The name of the consumable (one consumable per row). These items may include: tips, gloves, tubes, kits, labels, plate lids etc.)
2. The cost/unit (in local currency), plus specify what the unit is e.g. "Box of 100 gloves", "Box of 100 P200 tips"
3. How many units were used
4. State whether the goods were donated or purchased
5. If the good is donated, state what its market value would be.
6. Any relevant notes that can help clarify the market value attributed to the good in question.

Chapter

10

Chapter 10: Training and Certification

10.1 Trachoma Grading

The standard for this trial will be the World Health Organization simplified grading scheme, consisting of follicular trachoma (TF), trachoma intense (TI), and trachomatous scarring (TS). Field experience consists of graders observing eyelids together and agreeing on the grade, followed by one on one with Dr. Lietman, Keenan, and/or Gaynor, discussing eye lids clinical signs. When trachoma graders are sufficiently standardized, then we will have a formal trial, where 50 children are all graded by each grader, masked to the grades of the others. At the end, the score sheets are entered and kappa assessed for each grader compared to Dr Bailey.

If the kappa is 0.6 or greater, then the grader is certified for the trial. If the kappa is worse, then the grader must do more training to achieve a better agreement. No grader can be part of the data collection for the trial unless certified.

If, during the course of the trial itself, the review of photographs indicates that a grader is drifting, and the kappa for his clinical grades against his photogrades as read by Dr Bailey is less than 0.6, then the grader receives suspension of certification subject to re-training. The re-training can be done in country by another grader who is still certified. Once that grader and the suspended grader have carried out a trial in masked fashion, and agree with kappa =0.6, then the suspended grader can be reinstated provisionally. At the next clinical grading session, he must be certified again by Dr Bailey's review of the grades of the photographs compared to the clinical grades.

10.2 Laboratory Specimen Handling

The standard for this trial are the protocols developed and used by UCSF. During the standardization workshop, Dr Lietman's team will conduct training on appropriate laboratory procedures. A session where the protocol is described and details provided will be held. This will be followed by a field session where those who will be taking specimens will be observed for proper technique, from opening the packages, swabbing the eyelid and inserting the swab in the vial, and labeling. Open discussion of technique will take place for the first and possibly second attempts. The lab technician will need to perform properly on two eyelids to be certified for the trial. No lab technician can be part of the data collection for the trial unless certified.

Throughout the trial, air swabs are taken to check for possible field contamination. If any of the swabs are positive, then the lab technician receives provisionally certification, subject to review

by another, certified, lab technician in country. Once re-training is accomplished, the lab technician is re-certified, subject to review again of his field control swabs.

10.3 Photography

Each person who will be doing photography for the trial will receive training from Drs Bruce Gaynor, Tom Lietman, or Jeremy Keenan on the proper use of the handheld Nikon D series with macro lens to achieve gradable images of the upper lid. Training will consist of orientation to the camera, its parts, and care of the camera. Procedures will be taught that include shooting the identification number first, labeling the photolog with the patient identification number, and downloading and storing images on a CD for transmittal to Dr Bailey AND to the study site investigator.

Participants will practice shooting images of each other's eyelids until acceptable images are obtained, while shooting within a few seconds. Once the trainers feel the photographers are sufficiently ready, they will take their equipment to the field and while the trachoma graders/laboratory technicians are practicing in their sessions with trachoma cases, the photographers will shoot images. (In the event that the photographer is the same person as the lab tech or trachoma grader, then the photographer will practice on his/her own cases).

Certification will be granted once photographers pass an oral test on camera care, trouble shooting, and procedure, and can submit three consecutive acceptable photographs, with ID numbers, from the field, downloaded onto a CD. Photographers who do not pass this certification cannot be used in the trial.

10.4 Study Forms

The standardization workshop will include a session on the study forms which will include practice session with other team members posing as head of households for census, and practice with census updates, mass treatment, and examination forms.

In the field, those who are doing trachoma grading will practice filling in the ocular forms, the cost forms, and the mass treatment forms. Those supervising census will practice on households in the village with census forms and updates. Standardization exercises on observations of a clean face, presence of a latrine, and observations of garbage and waste around the doorway will also be done. The team leaders will be expected to teach their census teams these observations once they have been standardized at the workshop.

10.5 Data entry

Data entry personnel will be trained on study forms, to understand details on the content and completion requirements for the forms. They will then attend sessions on data entry programs for the study that will cover the following topics:

- How to maneuver through the data bases
- How to do data entry for each form
- Double data entry

- How to troubleshoot problem forms
- When to send forms back to the field for resolution
- Meaning of error messages and warnings
- Saving data bases
- Copying data bases for back up and encryption
- Printing reports
- Signing off and proper storage of paper forms
- Tips on Access

The data bases will have a manual associated with them for use by the statisticians and data managers.

The data managers must be certified by Mr Dreger, lead programmer for JHU, on data entry of all forms. Certification will consist of successful entry of a set of practice forms, saving and copying data bases, and proper sign off on forms. Once certified, the data manager can then train their data entry team members. All certified data entry team members must have their names sent to the study site investigators and the Executive committee members. Only data entry persons who have been trained and certified will be permitted to do data entry for this study, and their initials will be recognized for data entry.

Chapter 11

Chapter 11: Data Management and Routine Reports

11.1 Overview

To ensure appropriate data management, one person must be responsible for the supervision of data collection, processing, and storage. The study coordinator will be responsible for the supervision of data collection at the field sites during baseline and subsequent follow-up visits. Once the team has finished the field work in a community, the study coordinator will supervise data entry and management at the central office in each study site. The study coordinator will have ultimate responsibility for regularly checking with each individual to evaluate data flow.

All study forms were created in English and they will be translated to the local languages if needed. The process of translating forms is a multi-stage process that requires a minimum of two individuals fluent in both English and the local language. One individual will translate all forms into the local language. Once the translation is complete, the forms will then be given to a second translator who will translate the forms back into English. Both translators will review the forms for cultural sensitivity and understanding, and will report any problems to the principal investigator. The newly translated English-language forms will then be compared to the original forms to determine any differences that arose during the translation process. If corrections need to be made, the principal investigator will meet with the first translator to discuss how the differences arose and what changes need to be made to correct these differences. The first translator will then make the changes to the local language forms and give them to the second translator, who will again translate the forms into English. This process will continue until all participants in the translation process are satisfied that the local language and English language forms are interpreted in the same manner.

11.2.1 Study Forms Preparation

11.2.1.1 CENSUS FORMS

At baseline, each census taker is given a set of forms for a village, and a list of household numbers unique to them. The numbers may begin with a unique letter, then sequential numbers. At subsequent census, a master Census Book is created with the most recent version of the census for that village, which is used to update the census with new arrivals and departures.

11.2.1.2 MASTER SURVEY LIST

An electronic copy of the original census document is an electronic copy of the original census document. Using an electronic copy of the census, 120 children are randomly chosen. These children are listed on a Master Survey List, containing the full spelling of each individual's name, as well as their age, gender, and household number. A new Master Survey List is generated for each village at each collection visit and is used for both mobilization (the day before collection is scheduled) and registration.

11.2.1.3OCULAR EXAMINATION FORM

The Examination Form is printed for each of the 120 children who will be part of the sample survey in each village. Labels are also pre-printed for placing on tubes, photolog books, and specimen shipping log books.

11.2.1.4 BLANK CENSUS UPDATE FORMS

Blank forms are brought each day to the field during surveys or mass treatment in case they are needed to update information on sample children or residents.

11.2.2 Daily Data Management Activities in Field

All completed Master Survey Lists, Census update forms, Ocular Examination Forms and, depending on the activity, mass Treatment or Census books will be given to the Study Coordinator at the end of each workday and stored in a safe, secure place for transport at the visit's conclusion. The Study Coordinator will check the forms for completeness and accuracy.

11.2.3 Data Entry

The treatment data collected in the field will be entered into a database at PNLCC in Niamey. A double entry on key fields, using the uniform Access data entry package created for each site, will be done to minimize data entry errors. The study coordinator will do the final editing. The collection fieldwork data will be entered into a database by Proctor Foundation's Database Manager, who will also oversee the work of the staff member helping to double-enter the data. The Proctor Database Manager will also enter all PCR results into the database.

11.2.4 Preparation of Data For Transfer To UCSF

11.2.4.1 DATA MANAGEMENT

All treatment and collection data will be entered within four working weeks of collection. Data will be entered and inconsistencies will be resolved by the respective study coordinator or, if necessary, by consultation with the appropriate data collector. Access to any patient information will be protected by a password, or locked in a secure storage room. All data will be backed up upon entry, and forms will be kept until the conclusion of the study.

11.2.4.2 RECONCILIATION OF DATA

Before data entry, the forms will be reviewed and cross-checked for consistency and completeness. If the forms are not filled out completely, the Study Coordinator will contact the

person responsible for completing the form to provide missing data or clarify any inconsistent data. The Study Coordinator is the only person who is authorized to add missing data or make any changes to the study forms. All changes will be made in a different color ink and initialed and dated.

11.2.4.3 SPECIMEN TRANSFER PROCEDURES

In accordance with the Roche COBAS AMPLICOR™ CT/NG protocol, swab samples taken in the field will be transported on ice in a closed, insulated container until arrival in San Francisco, where they will be stored at -80°C for later analysis. Samples will be imported in the USA as per the CDC Permit to Import/Transfer Etiological Agents or Vectors of Human Disease.

11.2.5 Preparation of Data For Transfer To LSHTM

Two separate data entry clerks at PNLCC in Niger will double enter cost-effectiveness data using the Access program. The double-entered data will be verified, and any differences in data entry will be checked against the paper forms. The study coordinator will send the draft dataset to Proctor for verification. After reviewing the dataset with the Proctor Database Manager, the Proctor study coordinator will transfer the final dataset to the representative at LSHTM.

11.2.6 Data Editing At the Coordinating Center

Through range checks, the data entry software ensures to a large extent that there are no inconsistencies or invalid collection data. Data will also be checked by the Database Manager for consistency and errors.

The treatment data collected in the field will be entered into a database at PNLCC. A double entry will be done to minimize data entry errors. The study coordinator will do the final editing. The collection fieldwork data will be entered into a database by Proctor Foundation's Database Manager, who will also oversee the work of the staff member helping to double-enter the data. The Proctor Database Manager will also enter all PCR results into the database.

11.2.7 Data Management Process

All completed Census Forms, Master Lists, Ocular Examination Forms, and Treatment Forms will be given to the PNLCC Study Coordinator at the end of each workday and stored in a safe, secure place for transport. The Study Coordinator will check the forms for completeness and accuracy.

- Master List and Census Forms: Forms have patient information/names and will be organized by the PNLCC Study Coordinator and stay in Niger, stored in secure, locked area.
- Ocular Examination forms: Should be immediately photocopied, with one copy stored with PNLCC, and the other transported to the Proctor Foundation for double data entry under supervision of the Data Management Specialist.
- Treatment forms: Treatment coverage calculated and recorded, forms organized and stored in secure place at PNLCC office in Niamey.

11.2.8 Periodic Reports

PNLCC/Proctor Study Coordinators will deliver periodic reports updating partners on each collection and treatment visit. Reports will be deliverable within 60 days of completion of the visit.

11.2.9 PCR Result Reports

All PCR results will be double-entered at Proctor within one week of obtaining results. Any inconsistencies will be resolved by the appropriate study coordinator (PNLCC or Proctor), and if necessary, by consultation with the appropriate examiner or lab personnel. Patient names will not be made available to UCSF personnel; individuals will be identified by ID number only. All data will be backed up upon entry, and forms will be kept in locked cabinets through the conclusion of the study.

11.2.10 Data Storage

At the data-coordinating center, the operator takes a backup copy of the day's entry onto the data server and a CD. The data in the main computer will be backed-up daily and there will be at least two sets of backup disks at any particular time. All the backup CDs will be kept in a place different from the computer center.

Chapter

12

Chapter 12: Quality Assurance Policies and Procedures

12.1 Overview

Several policies and procedures are built into this project in order to provide assurances of quality in the data collection, processing, and management, as well as assuring proper treatment of all participants. General QA procedures include:

- Intensive training sessions: All individuals involved in the study will undergo training and certification for all study procedures for which they are responsible.
- Development of standardized forms and procedures for completion of forms. This includes procedures for recovery of missing data and changing responses, if necessary, as described below.
- Monthly data editing, report generation, and reconciliation, with feedback to the clinical sites in Niger, and the laboratories by each Data Coordinating Center. The process of providing periodic feedback on missing data or incomplete or out of range data is critical to timely recovery, identification of problems, and remedial actions to ensure that problems are solved.
- Monitoring of quality of trachoma grading and laboratory procedures used in the field, and in the laboratory. The Data Coordinating Centers will review the adherence to randomization procedures, treatment administered, timely follow-up of villages on schedule, agreement among graders for trachoma assessment in collaboration with Dr Bailey, and laboratory evidence of contamination of specimen collection or inadequate specimens. If the review uncovers a problem, the CC will notify the Study Site Principal Investigator, as well as issue reports to the Executive Committee. The clinical sites will immediately institute retraining procedures, including suspending a staff member from performance of the task until adequate performance can be demonstrated.
- Within each clinical site, the project directors have planned bi-weekly meetings of the census, survey, treatment, and the Data Entry Teams, to review reports and study progress, and address problems identified by the Data Coordinating Center.
- Masking of survey team as much as possible, and certainly the laboratory personnel, to treatment randomization. This procedure avoids bias in detection of infection, and determination of outcome.
- Active oversight by the Project Director in the field at the commencement of each study visit. The project directors will ensure the next phase of each part of the project proceeds smoothly, the data flow is steady, and the feedback for the new phase is in place.
- Site Monitoring with 6 monthly site visits by the Study Site Principal Investigator. These will focus on adherence to protocols, review of team meetings, and resolution of any issues.

- Creation of a paper or electronic trail to any data modifications and limiting access to the main database. Extensive back up procedures that protect the quality of the database. These procedures help ensure the integrity of the database.
- The Executive Committee will meet every six months to monitor, across sites, study progress, address any issues arising from data quality monitoring, review safety reports and review consent procedures.

12.2 General Guidelines for Form Completion

A significant component of quality assurance for data collection is the proper completion of forms. The following rules apply when completing the forms:

- Complete forms in "real time". Do not fill in the forms at a later time, or more convenient time. Later completion has a high potential for recall bias, missing data, or wrong data. The form must be filled in while the child/family member is present. The most difficult form to accomplish this will be the mass treatment form, which may be completed over a period of days for the village. However, it is essential that all persons be accounted for in terms of treatment. In the event of missing data, the staff must NOT try to recall the missing treatment but re-question the family to capture the data. If this cannot be done, it is better to code the data as missing (se below).
- Use only black ink for initial forms completion
- Use only red ink for alterations/edits to original entries on forms, which must be initialed by the person making the change.
- Check to make sure that ID labels/data and **study** identifiers are on every page of every form. Forms that do not have this information are to be referred back to the study site project directors for clarification and review.
- Print all written responses
- Do not change units or re-phrase questions.
- Enter data in the units and number of digits prescribed on form. Do not record fractions. Use a decimal only if it is written on the form. Be sure to include a value behind a decimal point, if applicable.
- Right justify all numbers, entering leading and following zeroes where applicable.
- Left justify all letter codes, leave remaining spaces blank.
- Write pertinent comments in the margins or on the last page of the form.
- Review all responses for completeness, skip patterns and accuracy before signing off on a form.

12.3 Missing Data on Study Forms

In summary, procedures conclude with the person responsible signing off on missing items or forms or vials that all attempts to locate have been undertaken and the item is missing. This form is transmitted to the Coordinating Centers in order to update the Master File.

If some data within forms are missing and cannot be obtained when the form is reviewed, then an appropriate code is inserted in the empty data field to indicate missing or not applicable.

12.4 Changing Responses on Study Forms

In general, responses should not be changed unless there was a transcription error, or the discovery of incorrect data by one staff on a form prepared by another. In these cases, the following guidelines should be adhered to:

Preserve the audit trail, so changes can be traced from original response to the changed response. Often, this means nothing more than making corrections on the original form. In the case of responding to questions raised by the data entry clerks, be certain that the missing data/specimen forms are properly filled out and accounting made to site project directors.

Do not obliterate, erase or white-out incorrect response. To correct a response: draw 1 or 2 lines through incorrect response, write correct response next to or about it in red ink, put initials and date in the margin by the correction.

12.5 Oversight

The Project Directors at each site will be responsible for overseeing the initiation of all phases of data collection. Thus, together with the study site principal investigators, the project directors will be present at the start of each new phase of data collection, specifically the census, surveys, mass treatment, and new rounds of each. The Project Director will be responsible for ensuring that each type of visit runs smoothly, and will remain onsite until all individuals involved in data collection for that visit type are comfortable with the data collection procedures. They will also be certain that the Data Entry team follows the procedures for each type of data entry, is operating smoothly, and responsibly storing the forms.

Bi-weekly staff meetings of project staff during census, survey, and will be held to review progress in enrollment/follow-up, review outstanding problems with forms or data entry, and reconcile reports from the coordinating center. The staff can report field problems, issues in dealing with the villages, and other concerns that might arise. All staff can attend, including drivers.

12.6 Adherence to Procedures

The Data Coordinating Center for each clinical site will monitor adherence to the procedures for randomization, mass treatment administered, census and follow-up surveys, adherence to standards for trachoma grading and laboratory evidence of contamination of specimen collection or inadequate specimens. Mechanisms are in place to ensure that villages are randomized to each treatment group in the absence of bias by study staff; and the treatment assignment is not known to the laboratory personnel.

12.7 Quality Assurance for Field Trachoma Grading

The images taken will be organized into folders for each examiner by visit. The folders will be sent to Dr Robin Bailey for review and grading, as he is the standard to which all the field graders have been trained. He will grade each set of 50 digital images for quality of the image, using 2.5 magnification. The first observation is for grade-ability of the image. If more than 20% of the images are ungradeable for any one photographer, the clinical site will be notified, certification of photographer will be suspended and the photographer will have to be re-certified.

Dr Bailey will grade each gradable photograph with a clinical grade for trachoma, using the WHO simplified grading scheme. The grades will be transmitted to each study site data coordinating center, which will compare the grades with the grades assigned to the eyes in the field. Agreement will be assessed, and if agreement falls below 0.6 in any month, the reasons for disagreement will be discussed, and the set of photographs re-circulated to bring agreement to acceptable levels. If necessary, the survey will be interrupted while the study site lead investigator and the field grader re-grade photographs and undertake field work until agreement is re-established. Some of the disagreement could be due to differences between grading photographs and grading live eyes in the field, although we have shown very high concordance.

The quality assurance of grading in the field is a required report from each center that will be presented at six months to the executive committee for review and reported to the DSMC during their annual review.

12.8 Quality Assurance for Specimens

Once per year, a set of five positive and five negative specimens from each laboratory will be re-aliquoted and half the sample will be sent to the Proctor laboratory, masked as to the findings from the original lab. The Proctor lab will re-run the results, as will the lab of origin for confirmation of results, thus providing assurances of similar procedures across laboratories. There must be at least 80% concordance of results between the laboratory and the Proctor lab.

The following steps will be followed:

- Dr Lietman and the lab of origin will send their results to the study site CC.
- The CC will send a report of the concordance, with ID numbers of non-concordant results, to the lab of origin and to Proctor.
- If concordance is at least 80%, the CC will issue a one year quality assurance certificate to the lab, and to Dr Lietman for his records, and to the Executive committee.
    - If concordance is less, the CC will issue a provisional notice to the lab chief and Dr Lietman, who must work together to resolve the difference, including re-sending specimens until concordance is reached. Once concordance is reached, the quality assurance certificate is again issued to the lab and to Dr Lietman for his records, and to the Executive Committee. Note that some of the discrepancy may be due to issues with shipping, resulting in loss of positivity of specimens. To the extent possible the re-aliquoted specimens in the laboratory will be stored under conditions that mimic the shipping and re-storage so that conditions of the specimens are similar.

The Executive Committee will review yearly the quality assurance certificates for the laboratories. Data on continued quality assurance will be presented to the DSMC.

There is also the possibility of field contamination, which is checked by using the “blue air” specimens. Each laboratory will process these controls masked as to their status as controls. The CC for each site is responsible for reporting the positivity of the negative field controls to the study site investigators. If the rate is above 1%, then the investigators must take remedial action to correct field procedures to remove contamination. Data on field contamination results will be presented to the DSMC for each site by the CC.

Chapter

13

Chapter 13: Adverse Events

Azithromycin is being given to these communities under the auspices of the National Trachoma Control programs in Niger. They have determined, for programmatic reasons, that widespread coverage with azithromycin is an acceptable community risk for the benefit of eliminating blinding trachoma. Millions of doses of oral azithromycin have been distributed for trachoma, and reports of serious side effects are essentially non-existent. This may be due in part to minimal surveillance. In fact, where carefully monitored, there were actually fewer GI side effects after taking azithromycin compared to topical tetracycline, perhaps because azithromycin treats some cases of diarrhea. Azithromycin is generally well-tolerated. The most common side effects of azithromycin and erythromycin are diarrhea or loose stools, nausea, abdominal pain, and vomiting, each of which may occur in fewer than one in twenty persons who receive azithromycin. Rarer side effects include abnormal liver function tests, allergic reactions, and nervousness.

All community residents will be advised to alert the village health worker if they experience, within two weeks of mass treatment, a serious adverse event, defined as diarrhea, nausea and vomiting for more than two days, hospitalization for any cause, or death in a family member. All adverse events will be recorded on a copy of the census list left with the village health worker by the mass treatment teams. These events will be reviewed at the next six month survey, and entered into the adverse event data base for reporting to the Data and Safety Monitoring Committee. If, for any reason, the village resident needs further care, they will be referred to the nearest health center for examination and treatment, and the most appropriate action will be taken to provide immediate care, in accordance with the policies in Niger.

Chapter

14

Chapter 14: Ancillary Studies

14.1 Risk of Re-emergence: Pediatric Longitudinal Study

We need a sample which can be followed longitudinally over time to determine how disease or infection re-emerges, and the value of multiple exposures to treatment. For this, a longitudinal sample of all children is needed. The sample for the trial is a simple cross sectional and thus the sample members may change at each round (as they age, or new residents move in, or as a consequence of sampling) this sample cannot address the critical questions of infection, trachoma, and re-emergence within children by number of years of mass treatment. For this, we propose a longitudinal cohort of children in a wider age range, for whom we can follow age and cohort effects. The second sample will consist of a longitudinal cohort of children ages nine and younger who will consist of all children in communities which are a sub-set of the randomly selected communities. For the longitudinal sample, we will randomly select up to three of the communities in each arm in which we will follow all children age 0 to 9 years at baseline. This sample will be a maximum of 750 children per arm, and a minimum of 300 children. Each year we will re-census the population of the communities to enroll any new children ages less than nine years. In this way, we can monitor the effect of treatment on successive age cohorts who have experienced variable numbers of rounds, and coverage, of treatment. They will also be followed up at each visit scheduled for the random sample, and exactly the same procedures for trachoma grading and ocular specimens will be used. Within each arm, at least one community will have all children ages less than 9 years followed longitudinally. These children will also serve as the random sample at baseline of children ages 0-5 years.

14.2 Cost Effectiveness Study

The cost effectiveness analysis will be based on community cost estimates that we will calculate using the cost collection instruments described below. For each community, we will collect cost information regarding: all medical and non-medical personnel involved in the study and all consumables used. We will also include laboratory costs attributable to screening/treating each community. The totality of the equipment costs - such as vehicles and field equipment costs – and laboratory costs will be recorded aggregately and then allocated to each community according to the community population.

14.3 Anthropometry: Niger

In Niger, trained examiners will take anthropometric measurements, including height, weight, and mid-upper arm circumference (MUAC), according to WHO guidelines and recommendations for each child 5 years of age and under who is already being examined in communities randomly selected communities in arms A, B, C, and D from the study. These

measurements will be recorded at the 12 month study visit and the 36-month study visit. In each community, of children who are randomly selected for trachoma monitoring, 62 children will be randomly selected for anthropometry in efforts to obtain 50 children. If there are less than 62 children in a community, all children will be selected.

A lightweight measuring board will be used to measure the participant's height to the nearest 0.1cm (Shorr measuring board). The SECA 874 scale will be used to weigh infants and children to the nearest 0.1 kg (Seca Corporation, Hanover, MD). Infants and young children can also be weighed simultaneously with their parent or guardian by the unique "mother-baby" function (parent or guardian is weighed and then the infant or child while held by the parent). Body mass index (BMI) will then be calculated later and used to determine the nutritional status of participants as severe malnutrition (BMI <15.9kg/m2), moderate malnutrition (BMI = 16-16.9 kg/m2), mild malnutrition (BMI =17-18.4kg/m2) and normal (BMI> 18.5kg/m2) (WHO, 1995). The child's MUAC will be measured at the mid-point between the tip of the shoulder and the tip of the elbow, to the nearest millimeter using non-stretch MUAC tapes produced for clinical studies by Johns Hopkins University and interpreted according to WHO guidelines. Any child with severe acute malnutrition, defined as a weight for height Z-score <-3.0, or severe chronic malnutrition, defined as a height for age Z score <-3.0 will be referred to a local health clinic for care. All treatment expenses will be paid for by the study.

14.4 Blood Collection: Niger

In Niger, all children being examined for anthropometric measurements (as previously described) will also receive blood testing. A single finger stick of capillary blood will be collected for a thick blood smear to assess for malaria, for hemoglobin testing, and for dried blood spots to be stored for later testing. Blood will be collected by a gloved health worker using aseptic technique. Gloves will be changed between each patient. The fingerprick or heelstick site will be disinfected using an alcohol swab. Once the alcohol dries, the health worker will prick the finger or heel area with a disposable lancet, and subsequently apply moderate pressure approximately 1 cm behind the site of the puncture to obtain sufficient drops of blood. The lancet will be disposed of in a biohazard waste container. A piece of gauze will be applied to the puncture site until bleeding has stopped. A large drop of blood will provide five small spot on FTA cards used for malaria monitoring (FTA Elute cards,Whatman, Kent, UK) and several microliters of blood absorbed onto six small circular extensions of another type of filter paper (TropBio Pty Ltd, Townsville, Queensland, Australia) used for serological testing, labeled with the patient's random ID number. Both blood spot samples will be left to air dry for 5-10 minutes before being placed in separate small storage bags. Blood spots will be stored at room temperature in a locked room in the study coordinators' office. A drop of blood will be placed in the center of a labeled glass slide and spread around evenly with a wooden stick. The smear will be allowed to air dry, and then fixed in the field using 100% methanol for 30 seconds. Smears will be transported at room temperature each day to a diagnostic facility in the nearest town to the study area (Matameye). Within 24 hours of thick smear collection, the smear will be stained with 2% Giemsa stain for 30 minutes, and parasite density will be measured by a masked reader using a microscope at the diagnostic facility. Smears will be stored at room temperature in the study coordinators' office. A portable spectrophotometer (Hemocue, Anglom, Sweden) will be used for hemoglobin testing. A cuvette will be introduced into a drop of blood at the fingerprick or heelstick site, and held there until completely full. The cuvette will be will be placed in the Hemocue system, and disposed of in an appropriate biohazards waste container. In all cases, study subjects will be identified by their study random identification number. Any children with any level of malaria parasitemia, or severe anemia, defined as

hemoglobin <5 g/dl, will be referred to a local health clinic for care. All treatment expenses will be paid for by the study.
